# Discordance between morphological and molecular species boundaries among Caribbean species of the reef sponge *Callyspongia*

**DOI:** 10.1002/ece3.1381

**Published:** 2015-01-13

**Authors:** Melissa B DeBiasse, Michael E Hellberg

**Affiliations:** Department of Biological Sciences, Louisiana State University202 Life Sciences Building, Baton Rouge, Louisiana, 70803

**Keywords:** Gene tree, Porifera, species delimitation, species tree, spicule

## Abstract

Sponges are among the most species-rich and ecologically important taxa on coral reefs, yet documenting their diversity is difficult due to the simplicity and plasticity of their morphological characters. Genetic attempts to identify species are hampered by the slow rate of mitochondrial sequence evolution characteristic of sponges and some other basal metazoans. Here we determine species boundaries of the Caribbean coral reef sponge genus *Callyspongia* using a multilocus, model-based approach. Based on sequence data from one mitochondrial (*COI*), one ribosomal (28S), and two single-copy nuclear protein-coding genes, we found evolutionarily distinct lineages were not concordant with current species designations in *Callyspongia*. While *C. fallax*,*C. tenerrima*, and *C. plicifera* were reciprocally monophyletic, four taxa with different morphologies (*C. armigera*,*C. longissima*,*C. eschrichtii*, and *C. vaginalis*) formed a monophyletic group and genetic distances among these taxa overlapped distances within them. A model-based method of species delimitation supported collapsing these four into a single evolutionary lineage. Variation in spicule size among these four taxa was partitioned geographically, not by current species designations, indicating that in *Callyspongia*, these key taxonomic characters are poor indicators of genetic differentiation. Taken together, our results suggest a complex relationship between morphology and species boundaries in sponges.

## Introduction

Coral reefs are the most species-rich habitats in the ocean, but accurately quantifying their diversity is difficult due to the simple and plastic morphological characteristics of many of their inhabitants. This is particularly true for the major reef building taxa: corals, algae, and sponges. Many studies have found incongruence between morphological taxonomy and genetic lineages (defined here as a group of individuals with an evolutionary history distinct from other groups) with taxa being overly split (Forsman et al. 2010; Prada et al. 2014), conservatively lumped (Klautau et al. 1999; Andreakis et al. 2007; Blanquer and Uriz 2007), or a combination of the two (Pinzon and LaJeunesse 2011). Additionally, rates of nucleotide substitution in the mitochondrial genome of sponges and anthozoans are 10 to 100 times slower than for bilateral animals (Shearer et al. 2002; Hellberg 2006), which can make the barcode sequence cytochrome oxidase I (*COI*) uninformative at the species level (Neigel et al. 2007; Huang et al. 2008; Pöppe et al. 2010).

Sponges are a group that would particularly benefit from a model-based approach to species delimitation. The paucity of informative taxonomic characters in the Porifera makes morphological species delimitation difficult, and their long generation times and large effective populations sizes can lead to incomplete lineage sorting and gene tree/species tree discordance (Degnan and Rosenberg 2009). Recently, multilocus species delimitation methods that account for the ancestral coalescent process have been developed (Yang and Rannala 2010; Edwards and Knowles 2014) and employed empirically (Leaché and Fujita 2010; Harrington and Near 2011; Satler et al. 2013; Prada et al. 2014) but have not yet been applied to the Porifera.

Sponges are among the most diverse taxa on coral reefs. Their biomass surpasses that of corals and algae in the Caribbean (Rützler 1978) and is expected to increase as oceans become warmer and more acidic (Bell et al. 2013). Nutrient cycling by sponges allows coral reefs to persist in oligotrophic seas (de Goeij et al. 2013). Sponges promote biodiversity by providing refugia for many commensal invertebrates, particularly during critical juvenile or reproductive life history phases (Ribeiro et al. 2003; Henkel and Pawlik 2005; Richards et al. 2007), and also harbor a substantial biomass of diverse microbial endosymbionts, many of which produce secondary metabolites with pharmacological potential (Taylor et al. 2007). Despite their ecological and evolutionary importance, few studies have combined genetic and morphological data to determine lower level relationships in sponges.

Sponge species in the genus *Callyspongia* (order Haplosclerida) have a wide range of growth forms (i.e., tube, massive, and rope shaped sponges) and are phenotypically variable within species, which makes them an excellent model for answering questions about the correspondence of morphological and molecular species boundaries in the Porifera. Of the 182 *Callyspongia* species presently recognized worldwide, eleven occur in the Caribbean, drawn from two different subgenera (*Callyspongia* (*Callyspongia*) and *Callyspongia* (*Cladochalina*)) (Hooper et al. 2002; van Soest et al. 2014). The most common Caribbean species, *C. vaginalis*, has at least two recognized growth forms (fan and tube shaped, Humann and De Loach 2003; Zea et al. 2014) and varies in color and texture (López-Legentil et al. 2010). Originally described by Duchassaing and Michelotti (1864), the taxonomic status of *C. armigera*,*C. eschrichtii*, and *C. longissima* is uncertain. Wiedenmayer (1977) considers *C. armigera* and *C. eschrichtii* to be growth forms of *C. vaginalis*, while van Soest (1980) supports the species status of *C. armigera* and *C. eschrichtii*. *Callyspongia longissima* is currently described as a valid species, but it may be another growth form of *C. vaginalis* (Zea et al. 2014).

Previous phylogenetic results have shown the genus *Callyspongia* and the species *C. vaginalis* to be paraphyletic based on mitochondrial and nuclear ribosomal sequence data (Erpenbeck et al. 2007; Raleigh et al. 2007; López-Legentil et al. 2010; Redmond et al. 2011, 2013). López-Legentil et al. (2010) found sequences for *COI*, 18S, and 28S were identical for *C. vaginalis* and *C. fallax* specimens sampled in Key Largo, Florida. In an ordinal level phylogeny estimated by Redmond et al. (2011) using sequences from the 5′ end of *COI*, the *C. vaginalis* and *C. fallax* individuals from López-Legentil et al. (2010) clustered together, a *C. vaginalis* specimen from Itskovich et al. (2007) clustered with a sponge from a different suborder, the giant barrel sponge, *Xestospongia muta*, and a *C. armigera* specimen from Erpenbeck et al. (2007) clustered together with *C. vaginalis* samples from DeBiasse et al. (2010). These phylogenetic patterns may be explained by the slow evolution of *COI* in sponges, misidentification of specimens, phenotypic plasticity, and/or DNA contamination and motivate our multilocus, model-based comparison of molecular and morphological species boundaries in *Callyspongia* from photo-vouchered specimens (Supplementary File 1).

## Materials and Methods

### Sample collection and generation of genetic data

Sponge samples from seven of the eleven Caribbean *Callyspongia* species were collected from two locations within each of two regions: Long Key, Florida, and South Acklins Island, Bahamas, in the northern Caribbean and Bocas del Toro, Panama, and Utila, Honduras, in the western Caribbean (Table1). Our sampling aimed to maximize the number of nominal species collected in sympatry (from Florida) while also including samples from distant locations to examine intraspecific and geographic variation. Sponges were identified using the online Sponge Guide (Zea et al. 2014). Our identification of *C. eschrichtii* is somewhat uncertain. According to Zea et al. (2014), it has yet to be determined whether these specimens are *C. eschrichtii* or one of the *Callyspongia* species described originally by Duchassaing and Michelotti (1864) and now synonymized under *C. vaginalis* (Lamarck 1813). Therefore, to reflect this uncertainty, we adopted the convention of Zea et al. (2014) and refer to these samples as *C. ?eschrichtii*. Specimens of all species were photographed in situ (Supplementary File 1) before a tissue sample was collected and stored in 95% ethanol.

**Table 1:** Species, location, and sample size information for specimens used in this study. Photo vouchers are available in Supplementary File 1. Accession numbers for all specimens and genes are available in Supplementary File 2

Species (subgenus)	Location	Sample size
*Callyspongia* (*Callyspongia*) *?eschrichtii*	Long Key, FL	3
	South Acklins Island, Bahamas	3
*Callyspongia* (*Callyspongia*) *fallax*	Long Key, FL	9
*Callyspongia* (*Cladochalina*) *armigera*	Long Key, FL	7
*Callyspongia* (*Cladochalina*) *longissima*	Bocas del Toro, Panama	1
*Callyspongia* (*Cladochalina*) *plicifera*	Long Key, FL	5
*Callyspongia* (*Cladochalina*) *tenerrima*	Long Key, FL	2
	Sweetings Cay, Bahamas	1
*Callyspongia* (*Cladochalina*) *vaginalis*	Long Key, FL	8
	Bocas del Toro, Panama	2
	Utila, Honduras	2
*Callyspongia* (*Cladochalina*) *vaginalis* cryptic species	Bocas del Toro, Panama	2
	Utila, Honduras	2

Genomic DNA was extracted using the Qiagen DNeasy kit. We amplified the 5′ end of the *COI* gene (Folmer et al. 1994), the traditional barcoding region, and the 3′ end of *COI* based on evidence that this downstream region of the gene may be more informative for sponge taxa (Erpenbeck et al. 2006). We also amplified the D1 region of the 28S gene and two single-copy nuclear protein-coding gene regions in the macrophage expressed protein (*mep*) and filamin (*fil*) genes, in which *C. vaginalis* evolve 3.6 and 7.1 times faster relative to *COI*, respectively (DeBiasse et al. 2014). PCR primers for the *COI* gene were designed from mitochondrial alignments of sequences downloaded from GenBank (Table S1). Primers for the nuclear protein-coding genes and the 28S gene were obtained from DeBiasse et al. (2014) and Redmond et al. (2011), respectively.

PCR amplifications were conducted in 25 *μ*L reactions consisting of 2.5 *μ*L of 10 × buffer, 10 *μ*mol/L of dNTPs and each primer, and 0.25 units of One Taq™ DNA polymerase (New England Biolabs Inc., Ipswich, MA). Amplifications were performed in a Bio-Rad T100 thermocycler under the following conditions: an initial denaturation cycle of 3 min at 95°C, 2 min annealing at 47 to 50°C and 2 min extension at 72°C, followed by 38 cycles of 30 s at 95°C, 45 s at 47 to 50°C, and 45 s at 72°C with a final extension cycle at 72°C for 10 min. Samples were sequenced in the forward and reverse direction using BigDye chemistry v3.1 on an ABI 3130XL at the Louisiana State University Genomics Facility. Forward and reverse sequences were aligned and edited using Geneious v4.5.4 (Drummond et al. 2012).

We resolved nuclear alleles in heterozygous individuals using PHASE v2.1 (Stephens et al. 2001). Individuals with alleles that could not be phased probabilistically to a probability >85% (*mep*: 1 *C. vaginalis*;*fil*: 2 *C. armigera*, 2 *C. ?eschrichtii*, 1 *C. longissima*, 2 *C. vaginalis*) were removed from the dataset. Nuclear gene regions were tested for intralocus recombination using GARD and SBP implemented in Hy-Phy (Pond and Frost 2005; Pond et al. 2006). Recombination was not detected in any gene region. We found no evidence of intragenomic variation in the 28S gene for any individuals. Sequences from *Amphimedon compressa* (*COI*, NC_010201; 28S, JN178945) and *A. queenslandica* (*mep*, GCF_000090795) were used as outgroups. Sequences for all individuals and genes are available from the European Nucleotide Archive under accession numbers listed in Supplementary File 2. Kimura 2-parameter (K2P) genetic distances within and among species for all gene regions were calculated in MEGA v5.2.2 (Tamura et al. 2011).

### Parsimony network and single locus gene tree estimation

Parsimony networks and maximum-likelihood gene trees for each locus were constructed to determine the relationships among alleles within and among the *Callyspongia* species. Networks were estimated in TCS v1.21 (Clement et al. 2000) using the default settings and resulted in the most parsimonious connections among alleles at the 95% confidence level. Models of nucleotide substitution were determined in jModelTest v2.0.2 (Darriba et al. 2012) based on the AICc, and maximum-likelihood trees were estimated in PAUP* (Swofford 2003) with 100 bootstrap replicates. We also estimated a maximum-likelihood tree to compare the placement of sequences generated in this study with those previously published (Table S2). We downloaded from GenBank 5′ *COI* sequences available for the Caribbean *Callyspongia* species examined here and species from the genus *Haliclona* because Redmond et al. (2011) found this genus and *Callyspongia* to be paraphyletic. We also included specimens of a *C. vaginalis* cryptic species from Central America (Bocas de Toro, Panama, *n* = 2 and Utila, Honduras, *n* = 2) identified by DeBiasse et al. (*in review*). We determined the model of nucleotide substitution and estimated the gene tree topology as described above.

### Species tree estimation and species delimitation

*Callyspongia fallax*,*C. plicifera*, and *C*. *tenerrima* each formed distinct clades in all gene trees (see section 3.1), suggesting they are unambiguous species. However, *C. armigera*,*C. ?eschrichtii*,*C. longissima*, and *C. vaginalis* shared alleles for all loci (see section 3.1). Motivated by these findings, we used a model-based approach that estimates the species phylogeny in the face of gene tree discordance due to incomplete lineage sorting to test the hypotheses that *C. armigera*,*C*. *?eschrichtii*,*C*. *longissima*, and *C*. *vaginalis* represent distinct evolutionary lineages. We used *BEAST v1.7.3 (Heled and Drummond 2010) to infer the species tree for these taxa using different combinations of loci: (1) *mep*,*fil*, 28S, *COI* (all loci); (2) *mep*,*fil*,*COI*, excluding the multicopy 28S gene, which evolves under concerted evolution, to determine whether this violation of the coalescent model influenced species tree estimation; and (3) 28S and *COI*, excluding the nuclear protein-coding loci due to their low resolving power. For each run, two independent MCMC analyses were conducted for 50 million steps, sampling every 5000 steps. Convergence was determined by viewing the log files in Tracer v1.5. All parameters had effective sample sizes (ESS) >300. Treefiles were combined in LogCombiner v1.7.1 with a 10% burn-in, and the maximum clade credibility (MCC) tree for the combined file was calculated in TreeAnnotator v1.7.1.

Using the species tree estimated in *BEAST as a guide tree, we evaluated nodal support for competing tree topologies where lineages represented by the *Callyspongia* species were maintained or collapsed in BPP v2.2 (Yang and Rannala 2010). Two independent BPP analyses with identical priors were run for 500,000 steps, sampling every five steps after a 50,000 step burn-in. We specified priors for theta (2, 2000) and tau (1, 10) that represented small ancestral population size and older divergence times, a conservative combination of priors that should favor maintaining current species designations (Leaché and Fujita 2010).

### Statistical analysis of spicule morphology

To test for differences in spicule morphology among taxa, we measured spicule length and width in *C. armigera*,*C. ?eschrichtii*,*C. longissima*,*C. vaginalis*, and the *C. vaginalis* cryptic species (DeBiasse et al. *in review*) (Table2). A subsample of tissue containing both the endoderm and ectoderm was incubated in a 75% bleach solution. Once sponge tissue was dissolved, spicules were rinsed and re-suspended in deionized water. The length and width of 50 intact spicules were measured for each specimen using light microscopy and the image analysis program SlideBook v6 at the Louisiana State University Socolofsky Microscopy Center. We used a mixed model analysis implemented in SAS v9.4 to determine whether spicule length or width varied (1) among species within locations (Florida, Bahamas, Panama, and Honduras), (2) among locations within regions (north: Florida, Bahamas and west: Panama, Honduras), or (3) between regions (north and west).

**Table 2:** Results of the mixed model analysis of variance in spicule size (length and width). Bolded values are significant

	*P* values	
Pairwise comparisons	Length	Width
Between Regions		
North and west	**0.0001**	**0.0001**
Within Regions		
Florida and Bahamas	**0.0003**	0.1836
Panama and Honduras	0.1536	**0.0024**
Within Florida		
*C. vaginalis* and *C. armigera*	0.9999	0.9970
*C. vaginalis* and *C. ?eschrichtii*	0.9999	0.9711
*C. armigera* and *C. ?eschrichtii*	0.9999	0.9999
*C. ?eschrichtii* between Florida and Bahamas	**0.0076**	0.9227
Within Panama		
*C. vaginalis* and *C. vaginalis* cryptic species	0.9726	0.0700
*C. vaginalis* and *C. longissima*	0.9999	0.7831
*C. longissima* and *C. vaginalis* cryptic species	0.9981	0.3099
Within Honduras		
*C. vaginalis* and *C. vaginalis* cryptic species	0.4206	0.3287

## Results

### Parsimony networks and single locus gene trees

**Figure 1:**
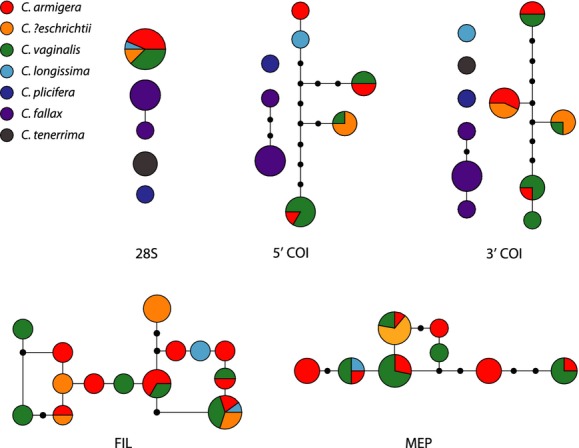
Unrooted statistical parsimony networks for each gene region with species represented by colors. Circles represent alleles with size proportional to frequency of the sequence. Small black circles represent possible but not sampled alleles, and lines between circles represent one mutational change between sequences.

Sequences from *Callyspongia fallax*,*C. plicifera*, and *C. tenerrima* did not connect to the TCS allele networks estimated using the *COI* or 28S genes (Fig.1). DNA sequences that fail to join a statistical parsimony network likely come from different species, and when enforcing the default 95% confidence limit, as we did here, the TCS method has a low false positive error rate (Hart and Sunday 2007). The mean K2P genetic distance between these three species and all others was between 10% and 28% (Table S3). C*allyspongia armigera*,*C*. *?eschrichtii*,*C*. *longissima*, and *C*. *vaginalis* all shared alleles in each of the parsimony networks (Fig.1). For all loci, the average mean K2P genetic distance among species for these four taxa (0–2.8%) was within the range of the mean K2P genetic distance within them (0–2.9%) (Table S3). In the 28S sequence alignment, *C. armigera*,*C*. *?eschrichtii*,*C*. *longissima*, and *C*. *vaginalis* shared one 6-bp indel and two 1-bp indels that distinguished them from *C. fallax*,*C*. *plicifera*, and *C*. *tenerrima*.

In the 28S gene tree, *C. fallax*,*C. plicifera*, and *C. tenerrima* each formed a monophyletic group corresponding to current species designations. *Callyspongia fallax* was also monophyletic in the *COI* trees, as was *C. plicifera* in the 3′ *COI* tree. We were only able to collect sequence data from one individual from *C. plicifera* and *C. tenerrima* from the 5′ and 3′ regions of the *COI* genes, respectively. These lone sequences did not cluster with any other sequences, and in their respective gene trees, they represented their own operational taxonomic unit. In each single locus gene tree, *C. armigera*,*C. ?eschrichtii*,*C. longissima*, and *C. vaginalis* clustered together within a single clade and the subgenera *Callyspongia* (*Callyspongia*) and *Callyspongia* (*Cladochalina*) were not monophyletic (Figs S1–S5). In a sequence alignment with no missing individuals, the 3′ region of *COI* had slightly higher haplotype (0.871) and nucleotide diversity (0.125) than the 5′ region of *COI* (0.840, 0.120). However, the 5′ and 3′ *COI* regions had the same gene tree topology (Figs S1 and S2), so for the *BEAST and species delimitation analyses, we concatenated these loci.

### Multilocus species tree and species delimitation analysis

**Figure 2:**
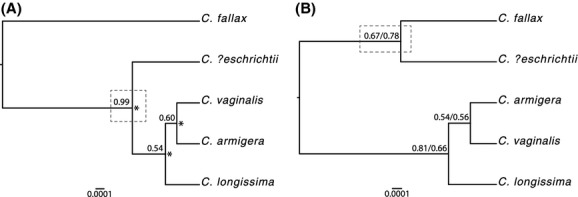
Species trees estimated in *BEAST. Dashed boxes indicate the change in position of *C. ?eschrichtii* between trees inferred from two sets of data. (A) Species tree topology estimated using 28S and *COI* gene regions. Posterior probabilities from the *BEAST analysis are listed at the nodes. Collapsed nodes in the highest probability BPP species delimitation model are indicated with asterisks. (B) Species tree topology recovered in the analysis of dataset 1 (all loci) and dataset 2 (nuclear protein-coding loci and *COI*). Posterior probabilities for the topology based on dataset 1 are listed first, and posterior probabilities for dataset 2 are listed second. Scale bar represents mutations per site.

The species tree estimated in *BEAST using 28S and *COI* had the highest nodal support, and its topology was consistent with the relationships recovered in the maximum-likelihood trees and parsimony networks (Fig.2A). The species tree estimated using all loci had low posterior probability for all nodes and placed *C. fallax* and *C. ?eschrichtii* as sister to a clade containing *C. armigera*,*C. longissima*, and *C. vaginalis* (Fig.2B), in contrast to the relationships supported by the parsimony networks and the maximum-likelihood gene trees. When we removed the 28S gene and repeated the analysis with *COI*,*fil*, and *mep*, the resulting species tree topology remained the same (*C. fallax* and *C. ?eschrichtii* sister) and the posterior support remained low (Fig.2B). Although the 28S gene violates coalescent assumptions because it is multicopy and evolves under concerted evolution, the effective population size of this marker is reduced, thereby decreasing sorting time and increasing resolution. rRNA genes have been useful in estimating phylogeny in sponges (Redmond et al. 2013; Thacker et al. 2013) and many other taxa (Mindell and Honeycutt 1990; Hillis and Dixon 1991; Hamby and Zimmer 1992). Here we found that it improves support in the species tree analyses.

The results of the BPP species delimitation analyses indicated that *C. armigera*,*C. ?eschrichtii*,*C. longissima*, and *C. vaginalis* belong to a single evolutionary lineage. The model with highest posterior support (0.99) was one that maintained only the node splitting *C. fallax* and the other four *Callyspongia* species (Fig.2A). The posterior support for this node was 1.0, while the support for the node splitting *C. longissima* from *C. ?eschrichtii*,*C. armigera*, and *C. vaginalis* was 0.0012. There was zero support for the node splitting *C. armigera* and *C. vaginalis* and the node splitting *C. armigera* and *C. vaginalis* from *C. ?eschrichtii*.

### COI gene tree

**Figure 3:**
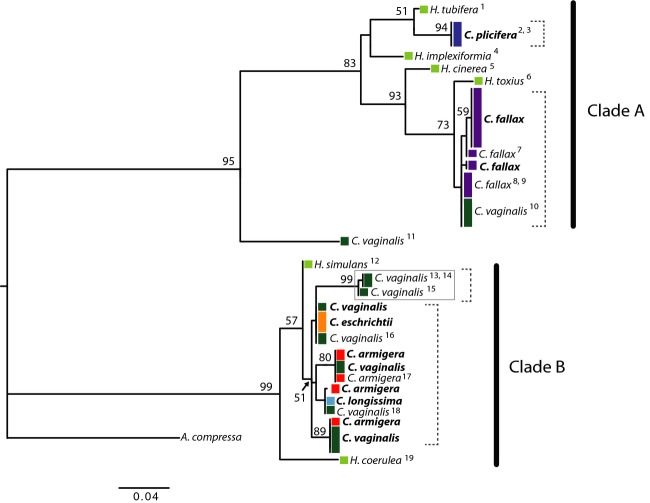
Maximum-likelihood tree estimated in PAUP* using *COI* sequences generated in this study (bold) and those downloaded from GenBank (with superscript numerals). Colored boxes indicate each species according to Fig.1, and height of the bar is proportional to the number of individuals with that sequence (e.g., one *H. tubifera* individual and three *C. plicifera* individuals). Support values from 100 bootstrap replicates appear at the nodes when greater than 50. Black bars show clades A and B recovered here and by Redmond et al. (2011). Dashed brackets indicate evolutionary significant units in *Callyspongia*. The gray box indicates the *C. vaginalis* cryptic species. The tree is rooted with *Amphimedon compressa* (accession number JN178945). GenBank sequences: ^1^Erpenbeck et al. (2007), EF519624; ^2^Kayal and Lavrov (2008), EU237477; ^3^Lavrov et al. 2008, NC010206; ^4^Erpenbeck et al. (2007), EF519624; ^5^Redmond et al. (2011) JN242198; ^6^Redmond et al. (2011), JN242206; ^7^Redmond et al. (2011), JN242192; ^8^Redmond et al. (2011), JN242193; ^9^López-Legentil et al. (2010), GQ415416–17; ^10^López-Legentil et al. (2010), GQ415412–15; ^11^Itskovich et al. (2007), EF095182; ^12^Redmond et al. (2011), JN242201; ^13^Erpenbeck et al. (2007), EF519577; ^14^DeBiasse et al. (*in review*), LK026489; ^15^DeBiasse et al. (*in review*), LK026544; ^16^Erpenbeck et al. (2007), EF519580–81; ^17^Erpenbeck et al. (2007), EF519578; ^18^Erpenbeck et al. (2007), EF519579; ^19^Erpenbeck et al. (2007), EF519619; see also Table2.

Sequences from the 5′ region of *COI* sampled in this study clustered with *Callyspongia* sequences deposited on GenBank by other authors (Fig.3). Although the genera *Callyspongia* and *Haliclona* are clearly defined based on morphology (De Weerdt 2002; Desqueyroux-Faúndez and Valentine 2002), the phylogenetic trees of Redmond et al. (2011) place species in two divergent clades that do not correspond to each genus. We recovered these two clades in our phylogeny (Fig.3). The sequence for *C. plicifera* generated here was identical to two other *C. plicifera* sequences obtained from complete mitochondrial genomes of this species (Kayal and Lavrov 2008; Lavrov et al. 2008). *Callyspongia armigera* and *C. vaginalis* sequences from Erpenbeck et al. (2007) and sequences from *C. armigera*,*C. ?eschrichtii*,*C. longissima*, and *C. vaginalis* and the *C. vaginalis* cryptic species generated here formed a clade (98% bootstrap support) excluding other closely related *Haliclona* species. There was high bootstrap support (100%) for a clade containing the *C. fallax* sequences generated here and those from López-Legentil et al. (2010) and Redmond et al. (2011). This clade also contained *C. vaginalis* sequences generated by López-Legentil et al. (2010). A *C. vaginalis* sequence from Itskovich et al. (2007) was sister to the clade containing *C. plicifera* and *C. fallax*. In the phylogeny estimated by Redmond et al. (2011), this sequence clustered with *Xestospongia*,*Petrosia*, and *Neopetrosia* species with high support (94%). The unexpected positions of *C. vaginalis* sequences (clustering with *C. fallax* and falling outside clades A and B) raise interesting questions about the possible biological (mitochondrial introgression) and technical (DNA contamination, misidentification due to phenotypic plasticity) explanations for unusual phylogenetic relationships in the Porifera and highlight the importance of thorough field notes, photo vouchers, and the integration of morphological and molecular data.

### Statistical analysis of spicule morphology

**Figure 4:**
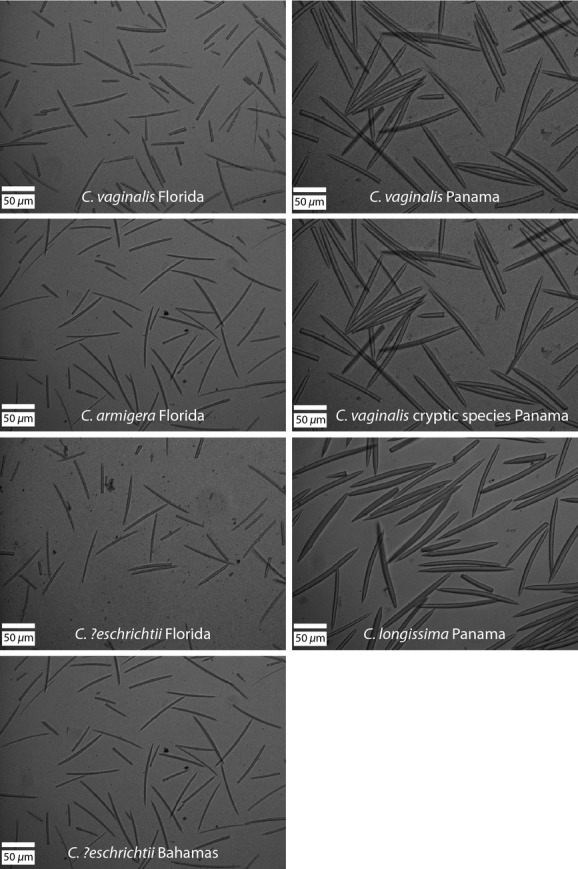
Photographs of spicules taken at 20 × magnification under light microscopy. Scale bars represent 50 micrometers.

A single spicule type (diactinal oxea) was present in *C. armigera*,*C. ?eschrichtii*,*C. longissima*,*C. vaginalis*, and the *C. vaginalis* cryptic species (Fig.4). Spicule length and width data for all species and locations are available in Supplementary File 3. Within species at each location, spicules fell into a single size class. The results from the mixed model analysis of spicule length and width indicated that variation in spicule size is better explained by geography than by current species boundaries (Table2). Spicule length differed between regions (north vs. west, *P *<* *0.0001) and between Florida and the Bahamas (*P *<* *0.0003), but not between Panama and Honduras (*P *=* *0.15). Within Florida, there was no difference in spicule length among *C. armigera*,*C. ?eschrichtii*, and *C. vaginalis* (*P = *0.9999 for all pairwise comparisons). There was no difference in spicule length between *C. vaginalis* and the *C. vaginalis* cryptic species in Honduras (*P *=* *0.4206), nor was there a difference among *C. vaginalis*,*C. longissima*, or the *C. vaginalis* cryptic species in Panama (*P = *0.9726–0.9999 for all pairwise comparisons).

For spicule width, regions differed (*P *<* *0.0001) as did Panama and Honduras (*P *=* *0.0024), but not Florida and the Bahamas (*P *=* *0.1836). Within Florida, there was no difference in spicule width among *C. armigera*,*C. ?eschrichtii*, and *C. vaginalis* (*P = *0.9711–0.999 for all pairwise comparisons), nor was there a difference among *C. vaginalis*,*C. longissima*, or the *C. vaginalis* cryptic species in Panama (*P = *0.07–0.9261 for all pairwise comparisons). There was no difference in spicule width between *C. vaginalis* and the *C. vaginalis* cryptic species in Honduras (*P *=* *0.3287).

## Discussion

The genetic analyses performed here support the species status of three *Callyspongia* species (*C. fallax*,*C. plicifera*, and *C. tenerrima*), but not four others (*C. armigera*,*C. *?*eschrichtii*,*C. longissima*, or *C. vaginalis*). We found extensive allele sharing among individuals of the latter four species (Fig.1), and the multilocus, model-based species delimitation analysis strongly supported collapsing these taxa into a single species (Fig.2). Although growth form varied among these nominal taxa (cluster of tubes: *C. vaginalis*; single tube: *C. longissima*; slender rope: *C. armigera*; solid mass: *C. ?eschrichtii*), spicule morphology did not vary among taxa within locations (Table2). Our genetic and spicule morphology results suggest that these taxa have been liberally split based on their overall shapes and should be considered growth forms of a single species, *C. vaginalis*, which was described by Lamarck (1813) prior to the other taxa (Duchassaing and Michelotti 1864).

### Molecular evolution among poriferan mtDNA, rDNA, and scnDNA and its implications for species delimitation

The molecular evolution of a genetic marker influences its utility in phylogeographic and phylogenetic inference. Here we combined sequence data from three types of genetic marker (mitochondrial, nuclear, and ribosomal) that evolve in different ways to delimit species boundaries in *Callyspongia*.

Slow rates of molecular evolution in the sponge mitochondrial genome have decreased its utility in phylogeographic and phylogenetic studies (Duran et al. 2004; Wörheide 2006; Pöppe et al. 2010). However, for some sponge taxa, particularly the Haplosclerida, mitochondrial loci appear to evolve less slowly and have been used successfully to estimate population structure and phylogenies (Duran and Rützler 2006; López-Legentil and Pawlik 2009; DeBiasse et al. 2010; Dailianis et al. 2011; Escobar et al. 2012). Variation in *COI* has also revealed cryptic species (Wulff 2006b; Blanquer and Uriz 2007; Pöppe et al. 2010; Xavier et al. 2010; Andreakis et al. 2012; de Paula et al. 2012), including *C. vaginalis* (DeBiasse et al. *in review*). Among the species examined here, *COI* genetic distances were up to 27% among taxa, suggesting variation at this locus is sufficient to distinguish among species in *Callyspongia*.

Although the 28S ribosomal gene violates coalescent assumptions because its many copies evolve under concerted evolution, this homogenization can reduce the effective population size of this marker, thereby decreasing sorting time and increasing its resolution for phylogenetic inference. Indeed, ribosomal genes have been useful in estimating phylogenies in sponges (Redmond et al. 2013; Thacker et al. 2013) and many other taxa (Mindell and Honeycutt 1990; Hillis and Dixon 1991; Hamby and Zimmer 1992). Sequences from 28S have strong phylogenetic signal for distinguishing among Haplosclerid species, and Redmond et al. (2011) and Redmond and McCormack (2008) demonstrated that indels in ribosomal genes were important synapomorphies in the Haploscleromorpha (marine Haplosclerids). Here we found the inclusion of 28S sequences improved support in the species tree analyses. Indels and segregation of SNPs in 28S further supported the species status of *C. fallax*,*C. tenerrima*, and *C. plicifera*.

In most bilateral taxa, nucleotide substitutions accumulate faster in the mitochondrial genome than the nuclear genome (Brown et al. 1979). In sponges and corals, however, this pattern is reversed (Eytan et al. 2009; DeBiasse et al. 2014). In *C. vaginalis,* single-copy nuclear protein-coding loci (including two used in this study – *fil* and *mep*) evolved up to 7.1 times faster than *COI* (DeBiasse et al. 2014). Nuclear protein-coding genes have been employed rarely in sponges (Sperling et al. 2009; Hill et al. 2013; DeBiasse et al. 2014), but given their fast rates (relative to *COI*) and the increasing ease of next generation data collection, nuclear loci hold promise for future phylogeographic and phylogenetic studies in sponges.

### Conflicts between morphological and molecular species boundaries in the Porifera

Mismatches between morphological species definitions and molecular data are common in sponges and fall across a spectrum of discordance. Of the seven Caribbean *Callyspongia* species we surveyed here, molecular species boundaries were concordant with morphologically defined species for three taxa: *C. fallax*,*C. plicifera*, and *C. tenerrima*. In the remaining four *Callyspongia* taxa, the relationship between morphology and evolutionary lineage was less clear. For example, in our investigation of population structure across the range of *C. vaginalis* (DeBiasse et al. *in review*), we found a genetically divergent cryptic species in samples from Honduras and Panama (Fig.3). Here we found no differences in spicule morphology between the cryptic species and sympatric *C. vaginalis* individuals, indicating conserved morphology masks genetic diversity. Cryptic species are prevalent in the Porifera, and phylogeographic studies, particularly on cosmopolitan species, often reveal deeply divided lineages among individuals that are morphologically indistinguishable (Reveillaud et al. 2010; reviewed in Xavier et al. 2010).

While we identified divergent lineages within morphologically identical specimens, we also found the opposite pattern. Although *C. armigera*,*C. ?eschrichtii*,*C. longissima*, and *C. vaginalis* differ morphologically in their shape, based on the genetic data we collected here, they belong to a single evolutionary lineage. The presence of multiple morphologies within a distinct genetic clade has been documented in other sponges (Loh et al. 2012) and in corals (Forsman et al. 2010; Budd et al. 2012; Prada et al. 2014). Interestingly, there was variation in spicule length and width among geographic regions but not among sympatric taxa. Such morphological differences in the absence of genetic divergence may be the result of biotic and/or abiotic factors acting on sponges. Predation pressure causes different growth forms in *Mycale laevis* (Loh & Pawlik 2009) and influences spicule density in *Anthosigmella varians* (Hill and Hill 2002). Water energy influences morphology in *Halichondria panicea* (Palumbi 1984, 1986) and *Cliona celata* (Bell et al. 2002), and wave action has been implicated in causing the fan-shaped morphology of *C. vaginalis*, which tends to be found in areas of high surge (VP Richards, personal communication; Humann and De Loach 2003). Maldonado et al. (1999, 2012) showed that spicule morphology and the presence or absence of a particular spicule type are influenced by seawater chemistry and the availability of nutrients such as silicic acid. Although microhabitat data were not collected as part of this study, we documented differences in spicule size among four different geographic locations, indicating environment plays a role in determining spicule morphology in *Callyspongia*. In the excavating genus *Cliona*, similar growth forms are repeated across genetically divergent lineages, suggesting these morphotypes represent developmental stages or adaptive phenotypes in response to habitat differences (Xavier et al. 2010; Escobar et al. 2012; de Paula et al. 2012).

### Divergent life history strategies between *C. armigera* and *C. vaginalis*

We found *C. armigera* and *C. vaginalis* to be indistinguishable genetically and based on spicule morphology. Previous work, however, found differences in growth rates and reproductive strategy between these taxa (Leong and Pawlik 2010, 2011). *Callyspongia vaginalis* generally devotes more resources to sexual reproduction, while *C. armigera* invests more in growth (Leong and Pawlik 2010). Accordingly, *C. vaginalis* relies on recruitment through larval dispersal, while *C. armigera* propagates primarily via asexual fragmentation. When larvae are present in *C. armigera*, they are smaller than those found in *C. vaginalis* (Leong and Pawlik 2011). Instead of marking inherent difference between species, this reproductive variation may constitute aspects of a suite of covarying plastic traits within a single species. For example, the slender rope morphology of *C. armigera* makes it more prone to asexual fragmentation, with rapid growth and reattachment after breakage (Wulff 2006a), potentially drawing on resources that could otherwise go to the production of larvae. *Callyspongia vaginalis*, on the other hand, contains more spongin fiber than *C. armigera* (Randall and Hartman 1968), which makes it more robust and less likely to fragment (Wulff 2006b), leaving larvae its primary mode of reproduction.

Alternatively, while we detected no evidence of genetic differentiation between *C. vaginalis* and *C. armigera*, we cannot rule out the possibility that there is divergence in other regions of the genome, perhaps those determining morphology or reproductive strategy. Such islands of divergence have been documented in other species (Martin et al. 2013; Parchman et al. 2013; Renaut et al. 2013) and are believed to be the first step in the process of speciation with gene flow (Wu 2001; Feder et al. 2012). For example, Martin et al. (2013) found genomic differentiation was strongest near loci determining divergent wing patterns in *Heliconius* butterflies, and in the passerine bird species *Manacus candei* and *M. vitellinus*, regions associated with reproductive isolation were scattered across the genome (Parchman et al. 2013). A genomewide examination of *Callyspongia* in conjunction with analysis of microhabitat data, transplant experiments, and reproductive crosses would shed light on whether differences in morphology and reproductive strategy are driven by environment or by divergent regions of the genome other than the ones surveyed here.

Sponges present a challenging, yet fascinating, system for testing hypotheses of species delimitation. The potential to untangle the combined influences of molecular evolution, environment, and life history on the evolution of sponge morphology and species boundaries will undoubtedly increase as more genomic data become available for this group (Riesgo et al. 2014).

## Supporting Information

**Supplementary File 1.** Photo vouchers: Photo vouchers and sample information for sponge samples used in this study.

**Supplementary File 2.** Accession numbers: A file containing samples size information and accession numbers for all genetic loci.

**Supplementary File 3.** Data for sponge spicule analyses: A spreadsheet containing the raw spicule length and width data.

**Table S1.**Primer sequences and expected amplicon size for gene regions used in this study. **Table S2.***COI* sequences downloaded from GenBank used in Figure 3. **Table S3.** (A–E) Mean Kimura 2 Parameter genetic distances within and among species for each gene region. Italicized values are those estimated within species. **Figure S1.** Maximum likelihood tree estimated in PAUP* using the 5′ region of the *COI* gene. **Figure S2.** Maximum likelihood tree estimated in PAUP* using the 3′ region of the *COI* gene. **Figure S3.** Maximum likelihood tree estimated in PAUP* using the D1 region of the 28S gene. **Figure S4** Maximum likelihood tree estimated in PAUP* using a portion of the filamin gene. **Figure S5.** Maximum likelihood tree estimated in PAUP* using a portion of the macrophage expressed protein gene.
