# Supplementary material for: Discordance between morphological and molecular species boundaries among Caribbean species of the reef sponge Callyspongia
Source: Ecol Evol. 2015 Jan 13;5(3):663–75. doi: 10.1002/ece3.1381 (PMC4328770; doi:10.1002/ece3.1381)
Supplement: Supplementary file 1 [file ece30005-0663-sd1.pdf]

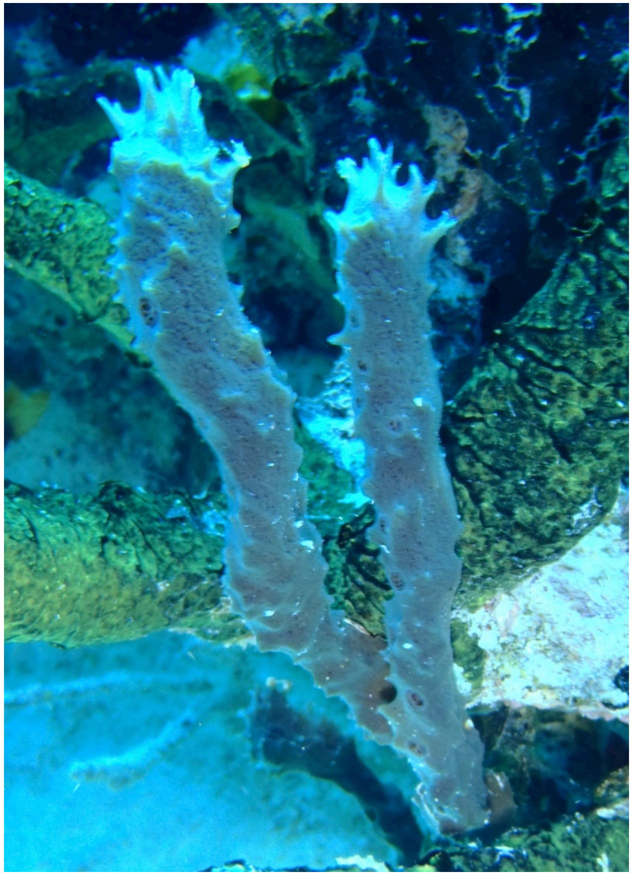

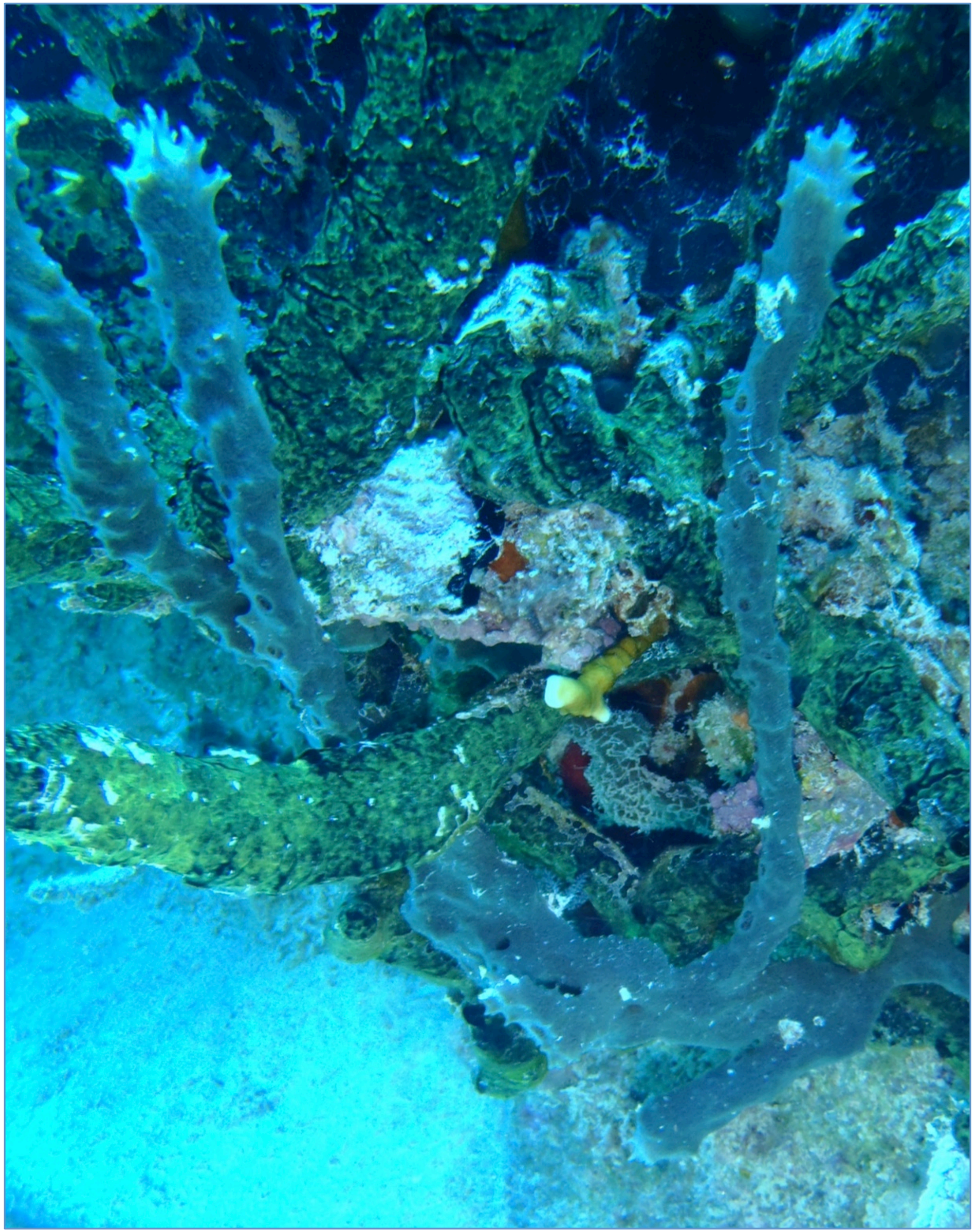

Callyspongia armigera
ARM07
N24° 44.016, W80° 49.590
Coffins Patch Reef
Collected on 03/29/13 by
KML staff

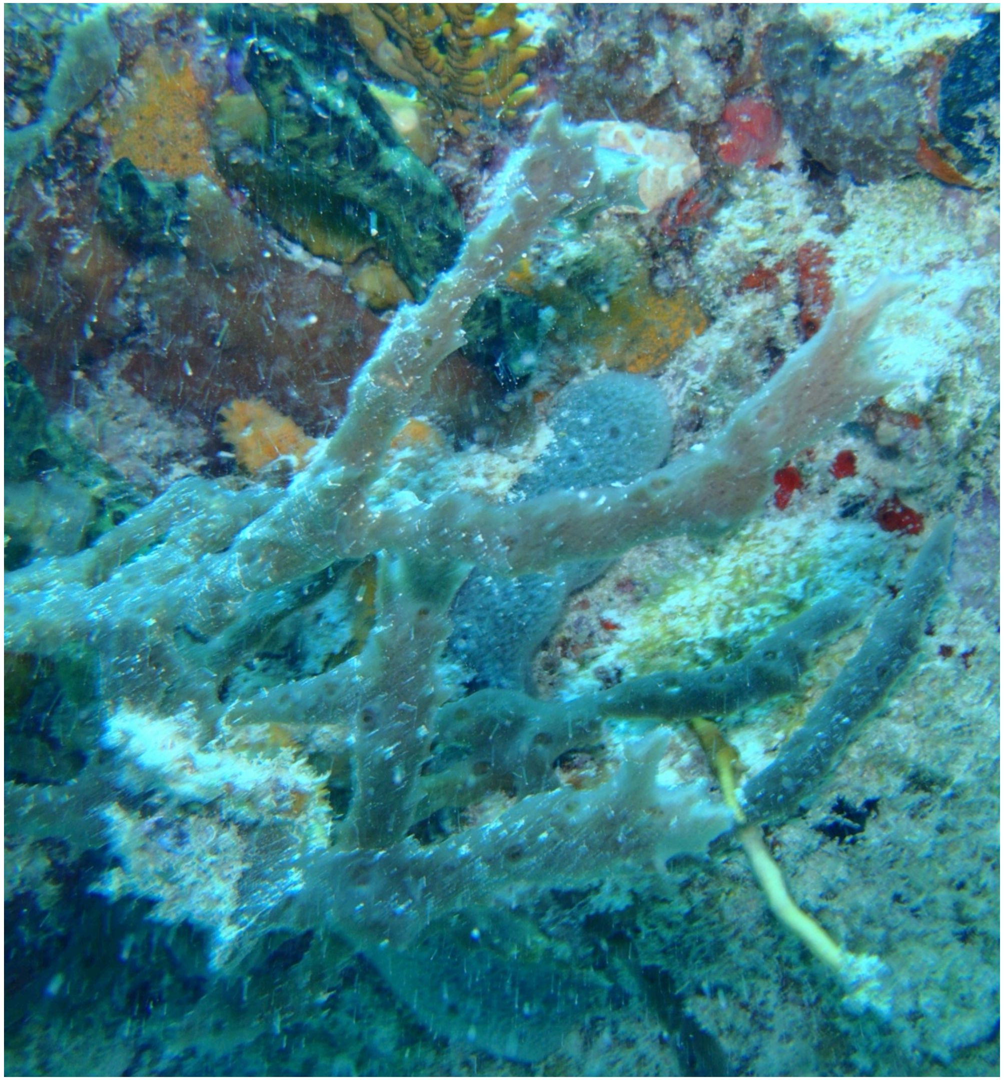

Callyspongia armigera
ARM08
N24° 44.016, W80° 49.590
Coffins Patch Reef
Collected on 03/29/13 by
KML staff

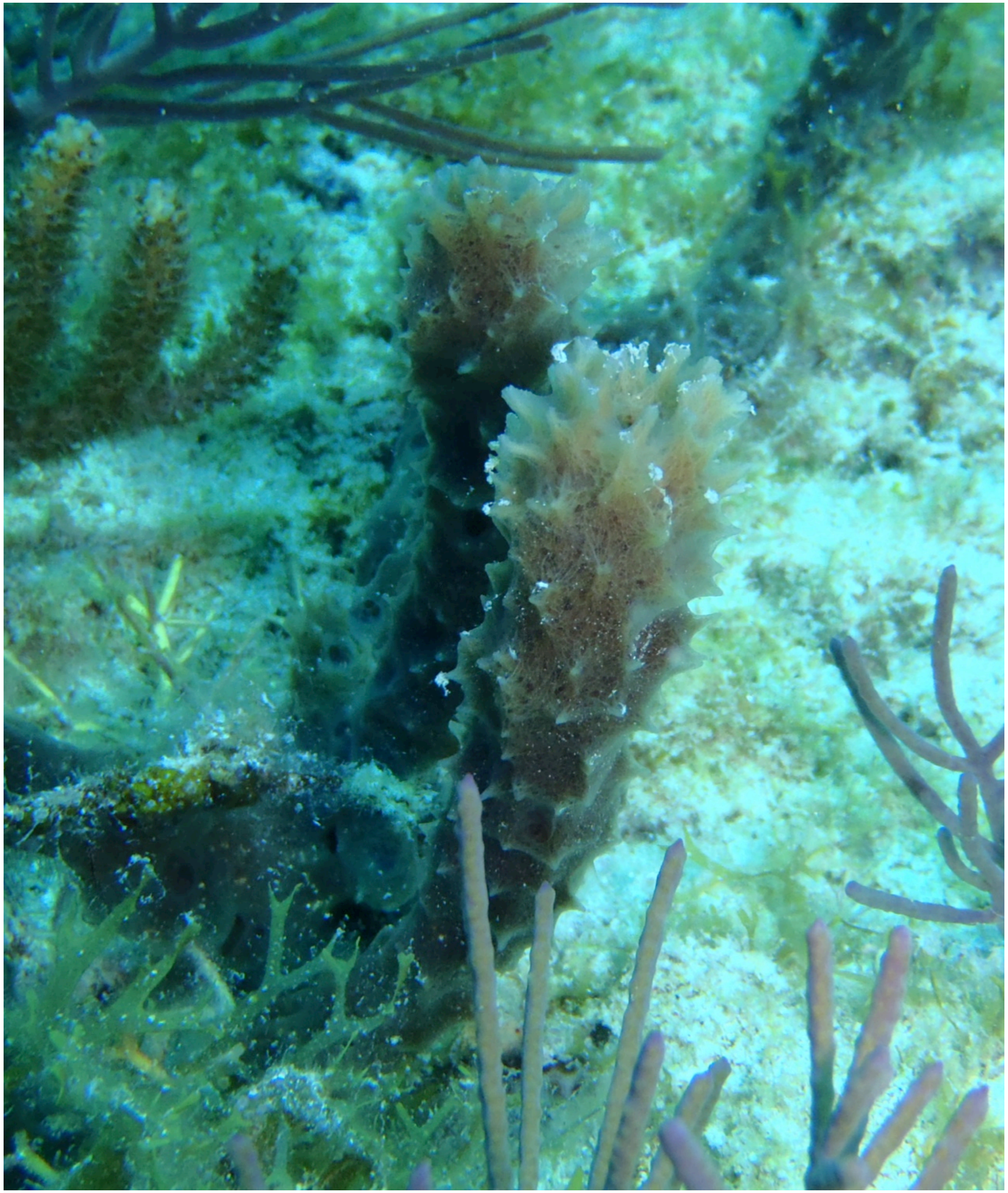

Callyspongia armigera
ARM53
N24° 45.953, W80° 45.239
Tennessee Reef
Collected on 04/02/13 by
KML staff

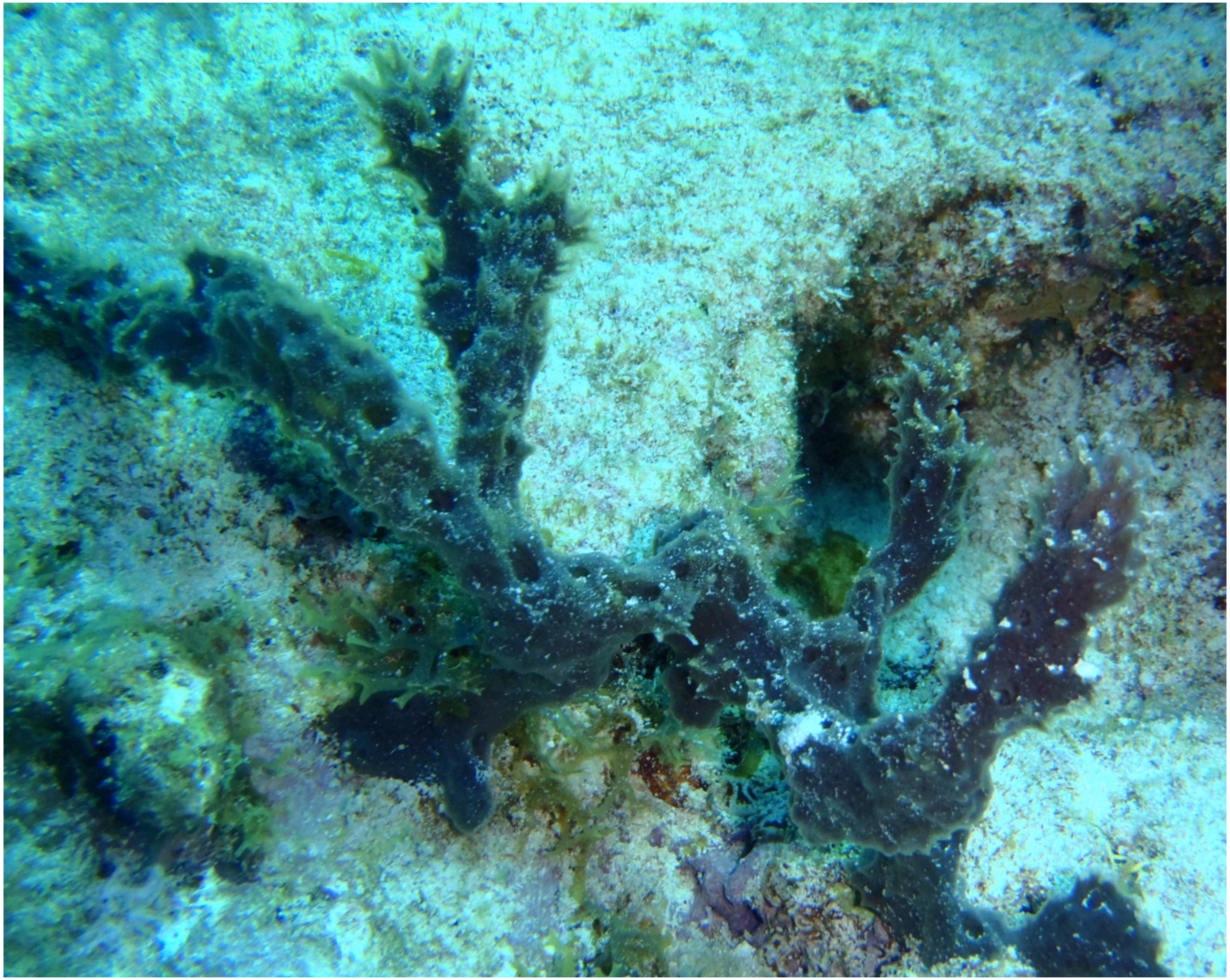

Callyspongia armigera
ARM54
N24° 45.953, W80° 45.239, Tennessee Reef
Collected on 04/02/13 by KML staff

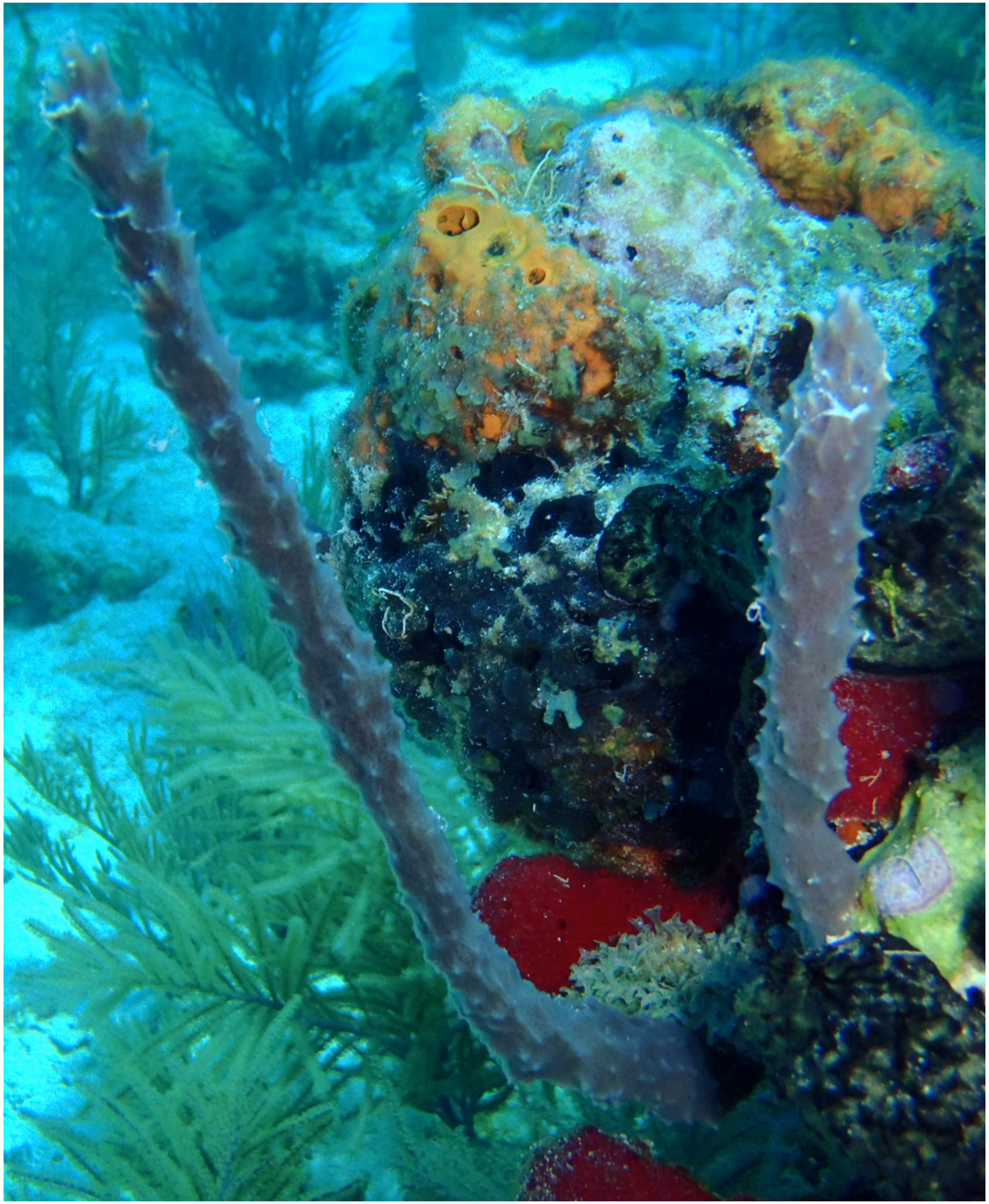

Callyspongia armigera
ARM55
N24° 45.953, W80° 45.239
Tennessee Reef
Collected on 04/02/13 by
KML staff

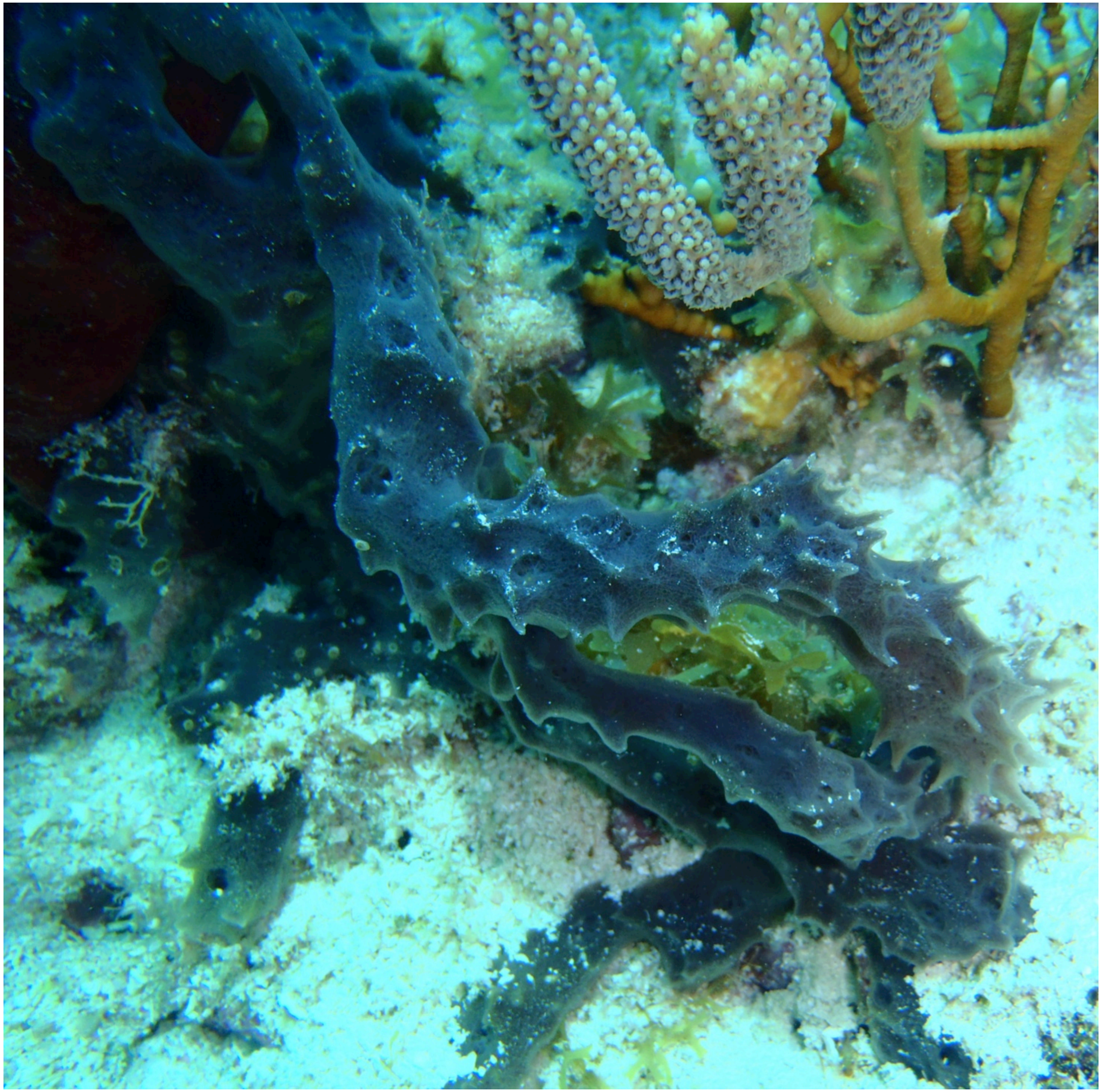

Callyspongia armigera
ARM56
N24° 45.953, W80° 45.239
Tennessee Reef
Collected on 04/02/13 by
KML staff

Callyspongia armigera
ARM57
N24° 45.953, W80° 45.239, Tennessee Reef
Collected on 04/02/13 by KML staff

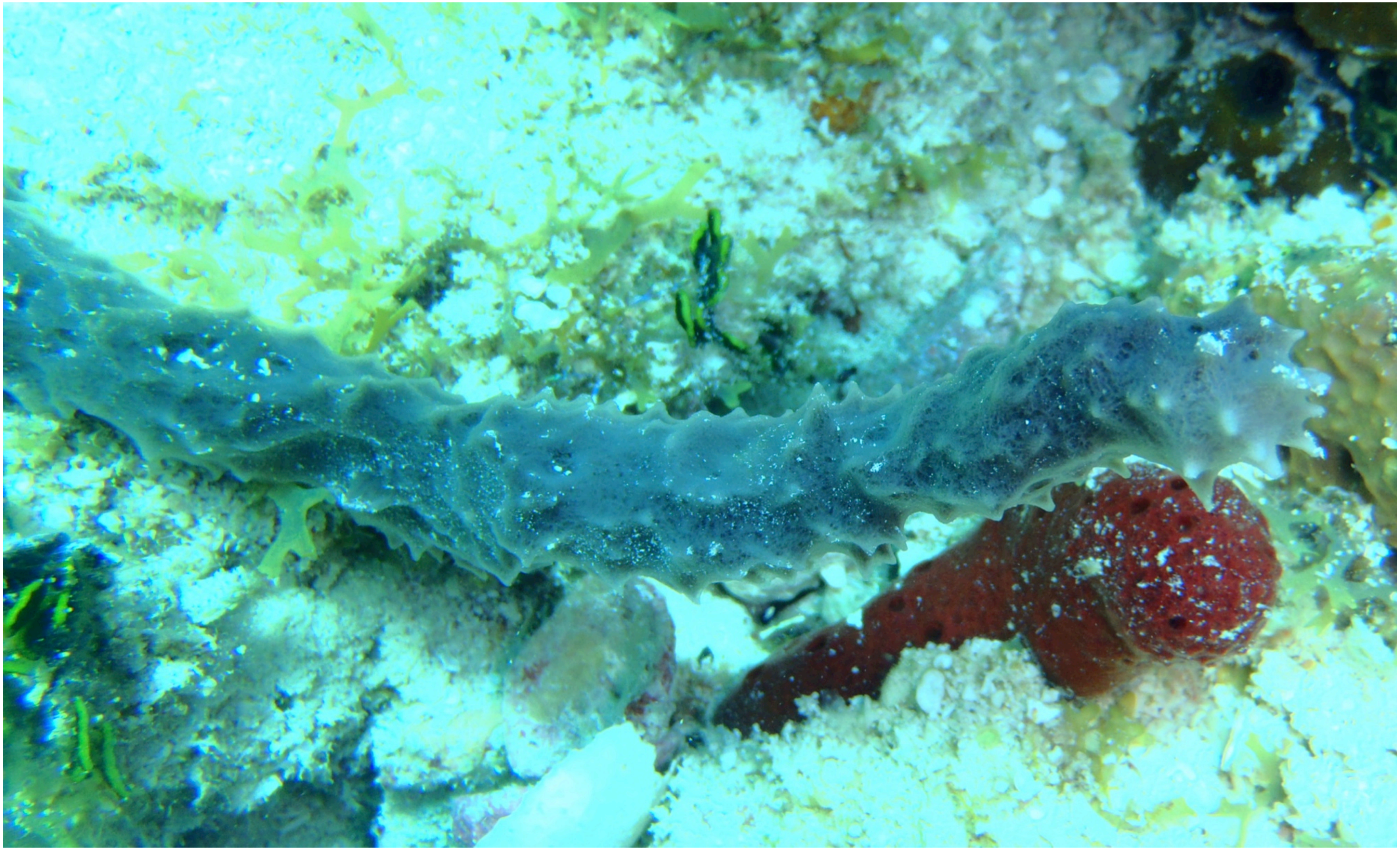

Callyspongia fallax
FAL12
N24° 44.016, W80° 49.590, Coffins Patch Reef
Collected on 03/29/13 by KML staff

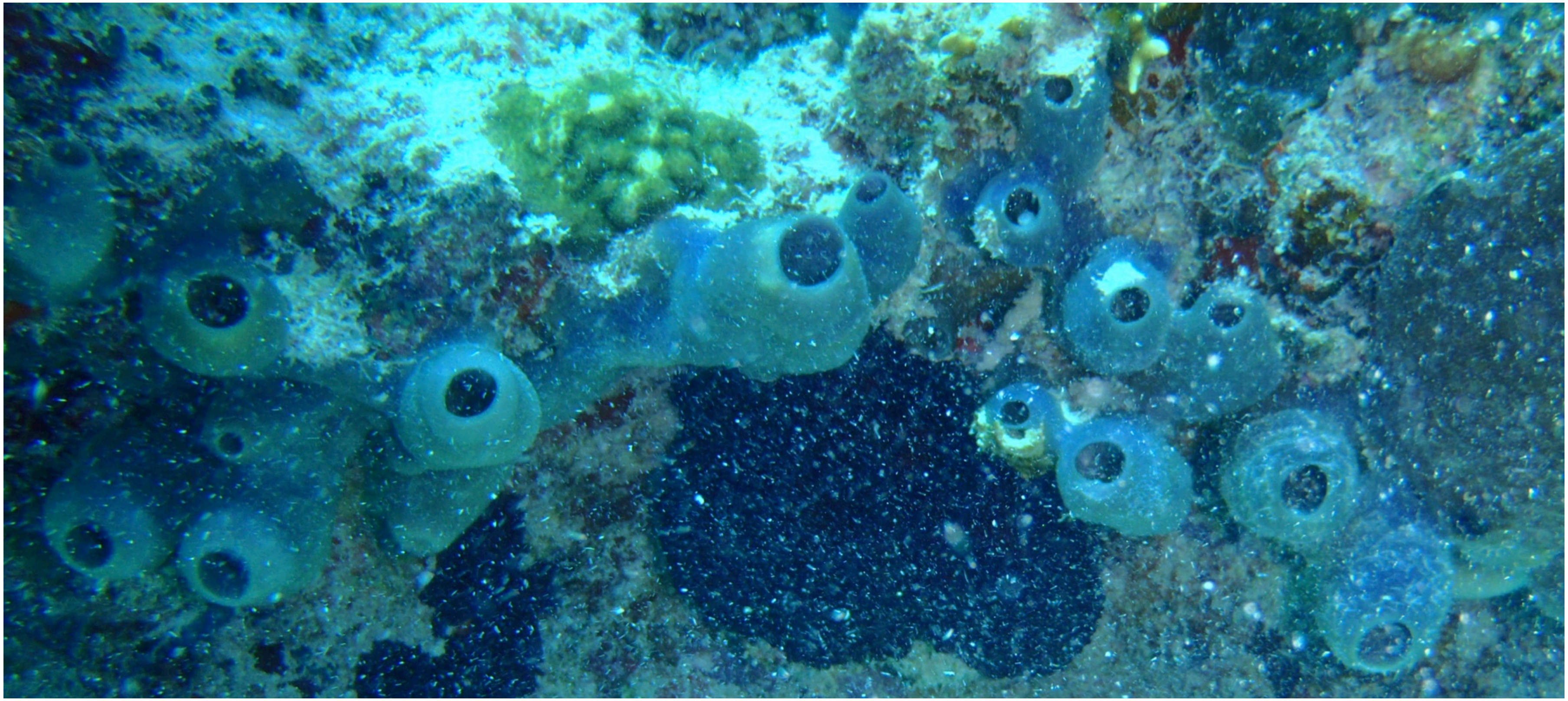

Callyspongia fallax
FAL13
N24° 44.016, W80° 49.590, Coffins Patch Reef
Collected on 3/29/2013 by KML staff

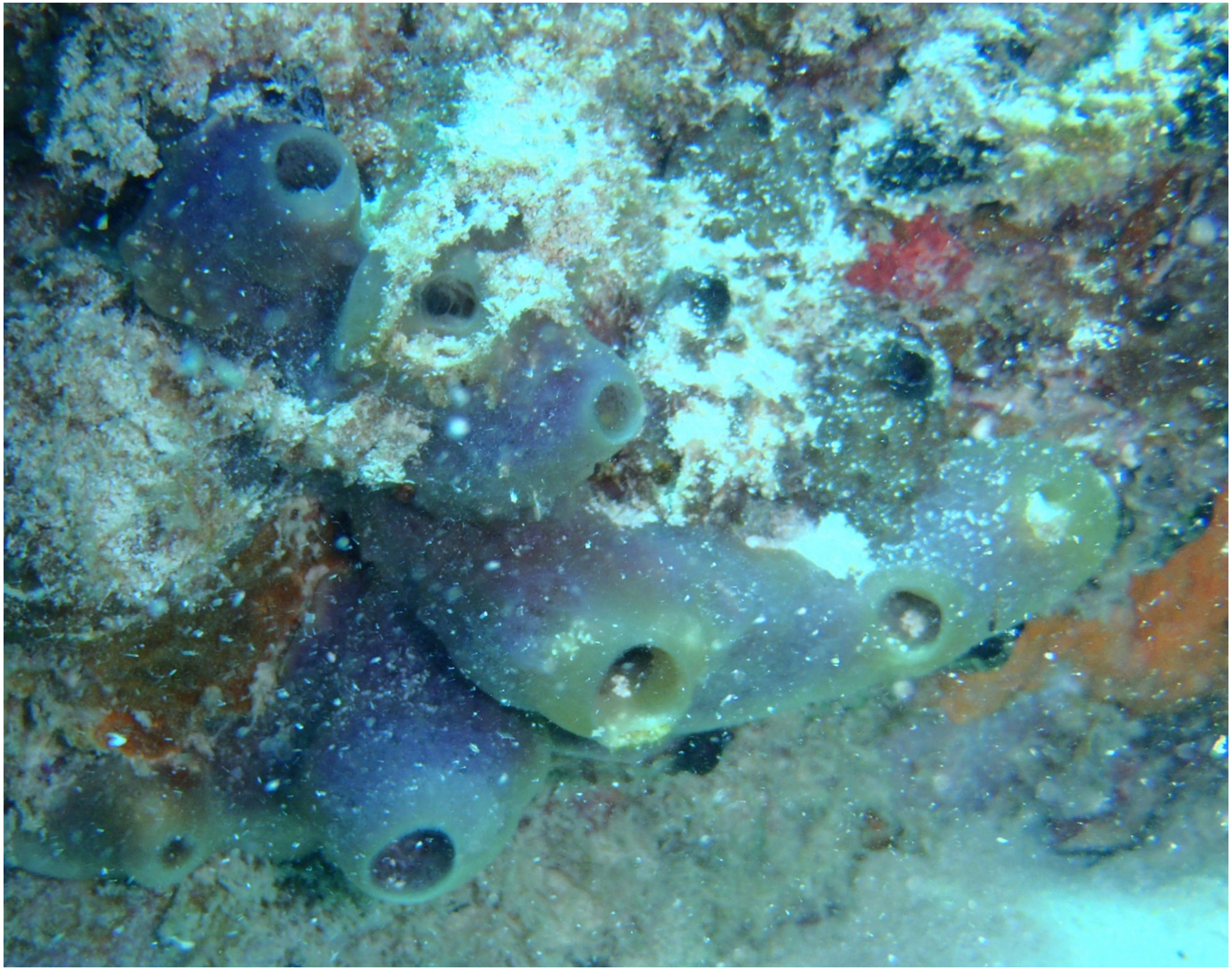

Callyspongia fallax
FAL14
N24° 44.016, W80° 49.590, Coffins Patch Reef
Collected on 03/29/13 by KML staff

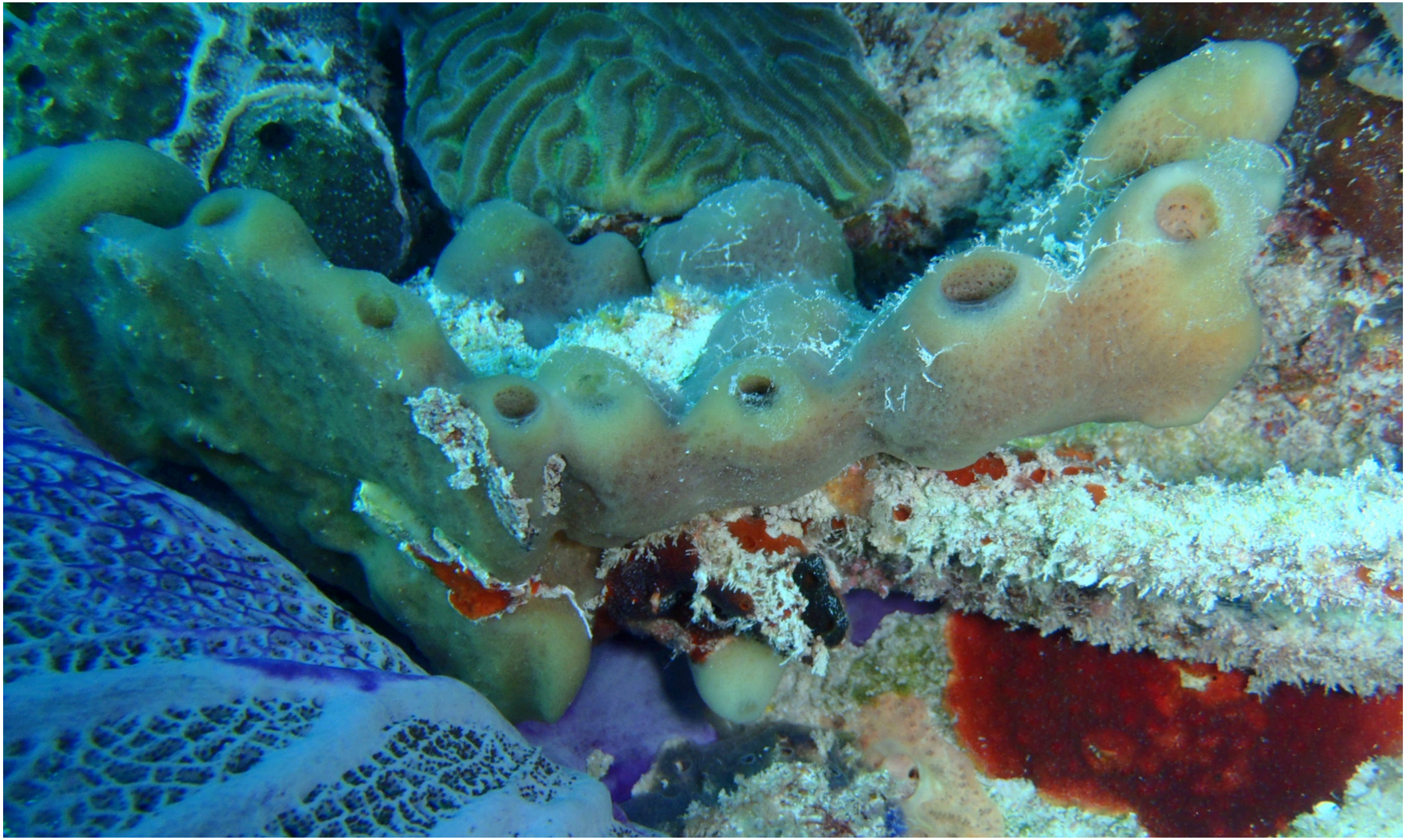

Callyspongia fallax
FAL15
N24° 44.016, W80° 49.590, Coffins Patch Reef
Collected on 03/29/13 by KML staff

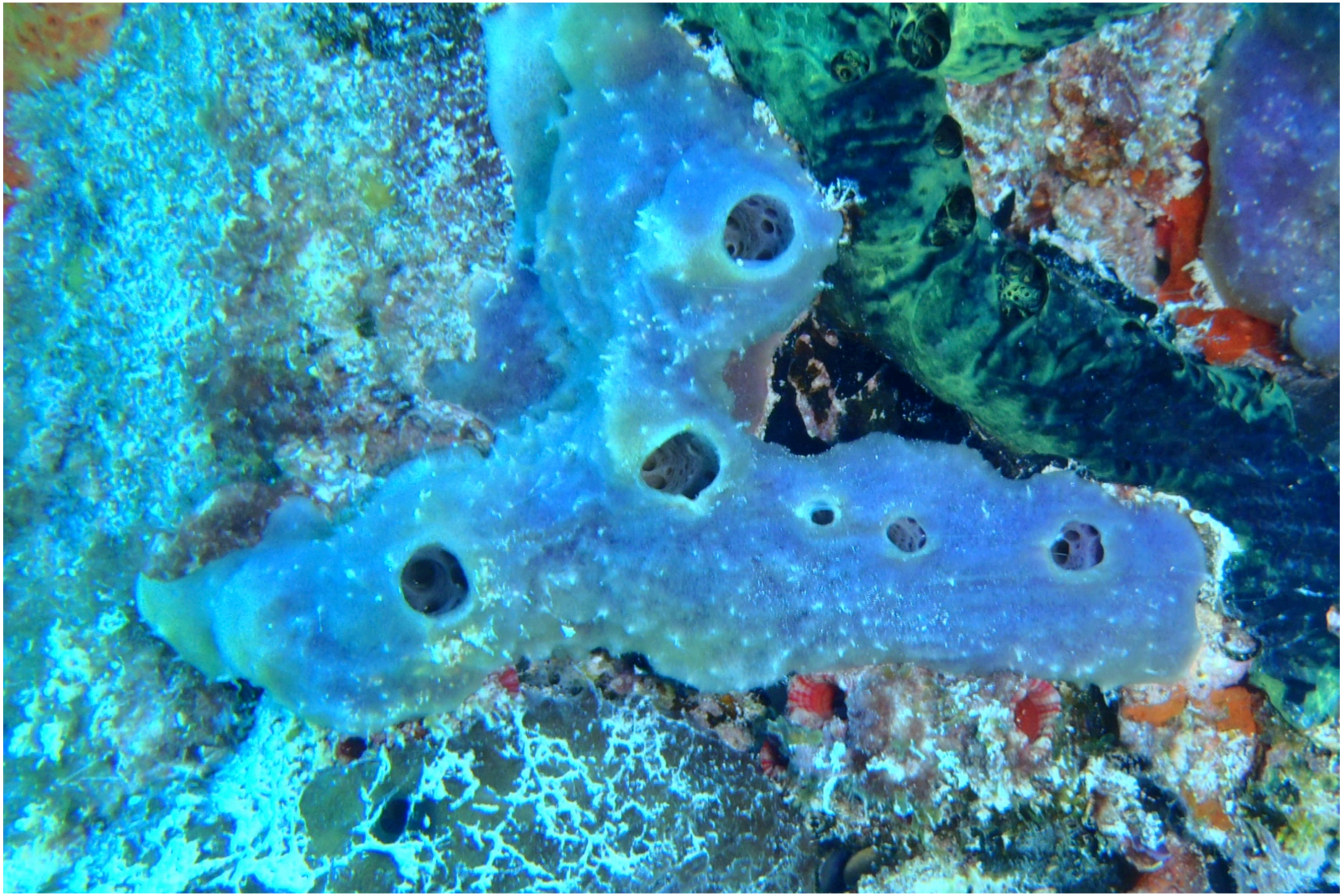

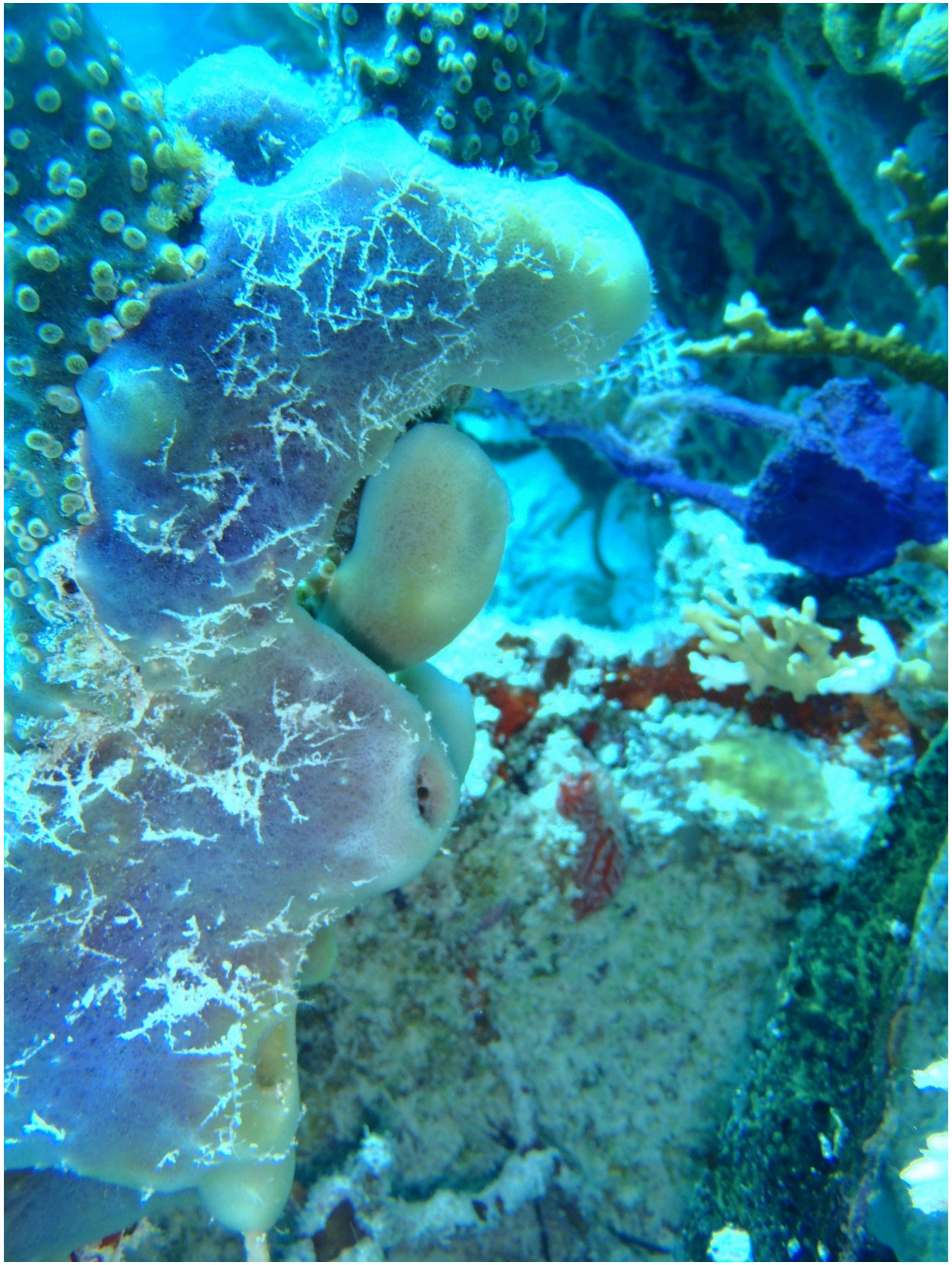

Callyspongia fallax
FAL16
N24° 44.016, W80° 49.590
Coffins Patch Reef
Collected on 03/29/13 by
KML staff

Callyspongia fallax
FAL17
N24° 44.016, W80° 49.590, Coffins Patch Reef
Collected on 03/29/13 by KML staff

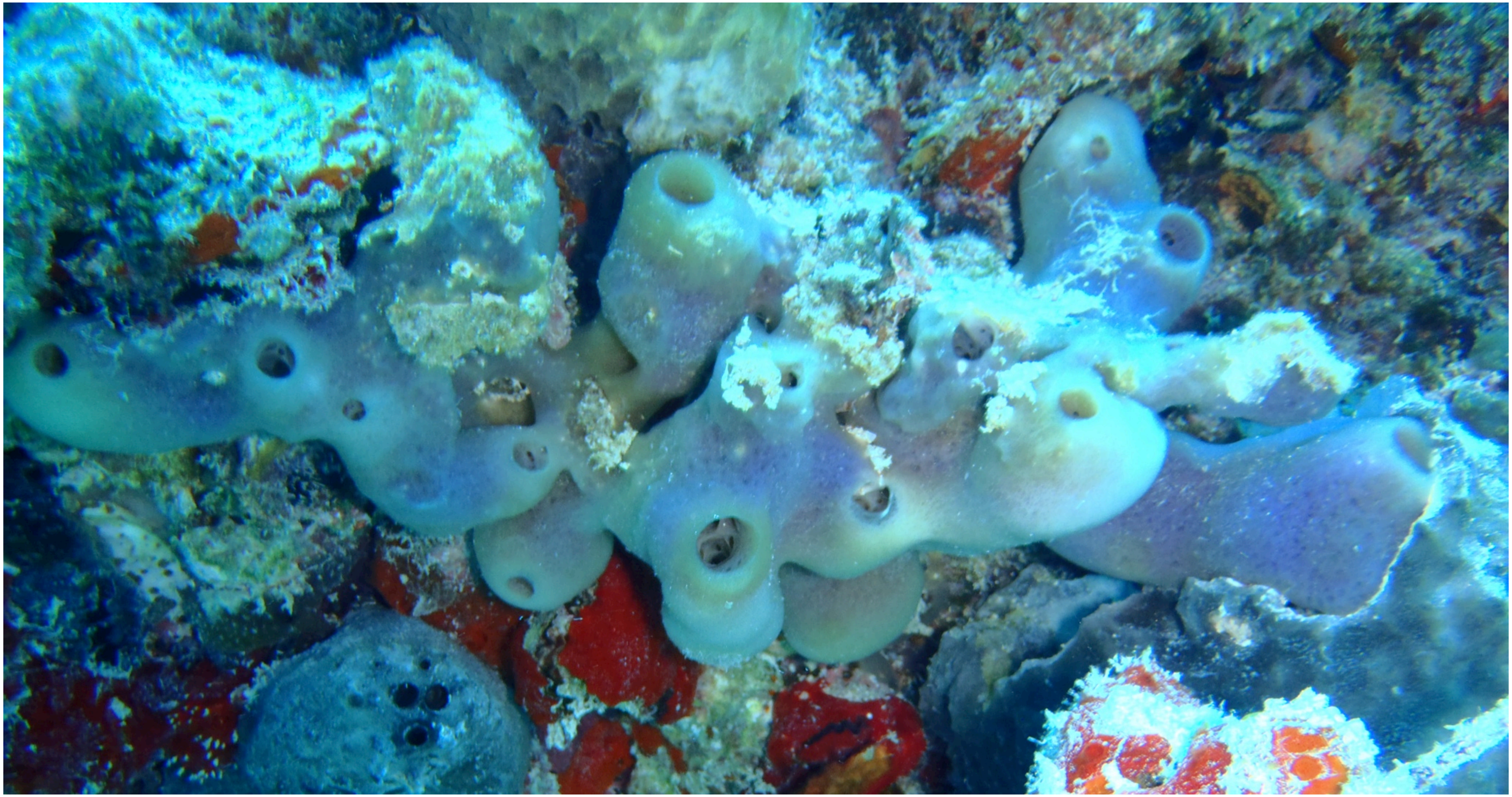

Callyspongia fallax
FAL18
N24° 44.016, W80° 49.590, Coffins Patch Reef
Collected on 03/29/13 by KML staff

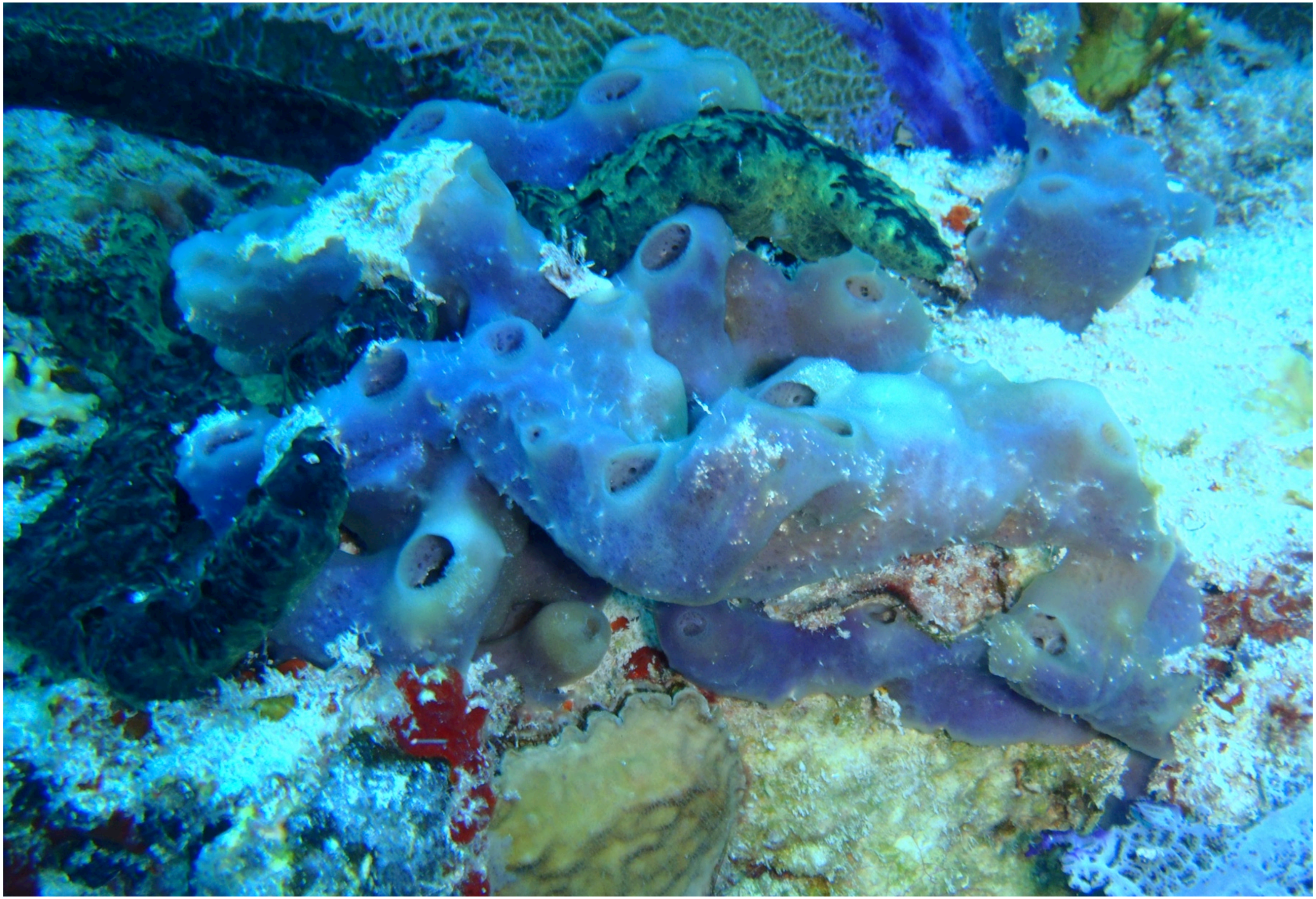

Callyspongia fallax
FAL19
N24° 44.016, W80° 49.590, Coffins Patch Reef
Collected on 03/29/13 by KML staff

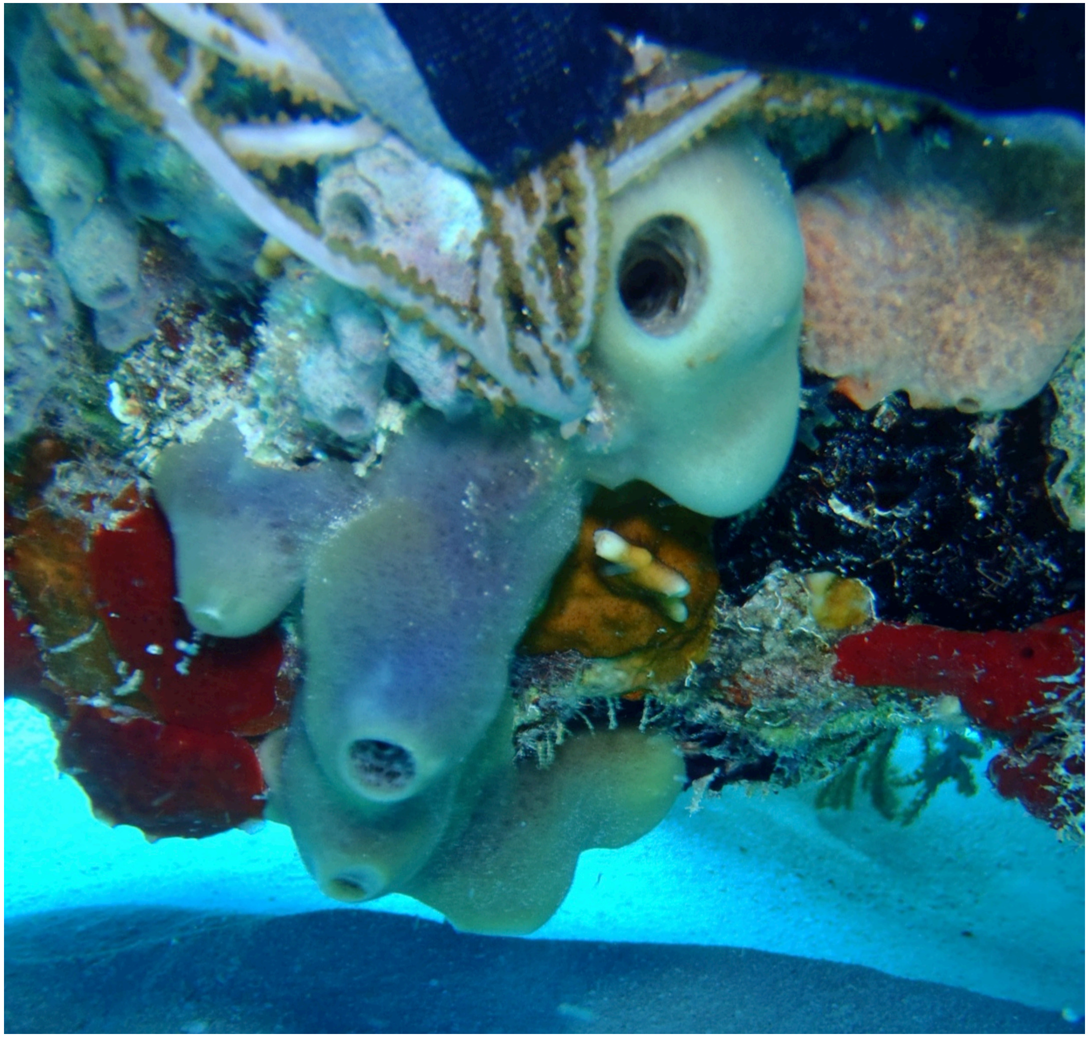

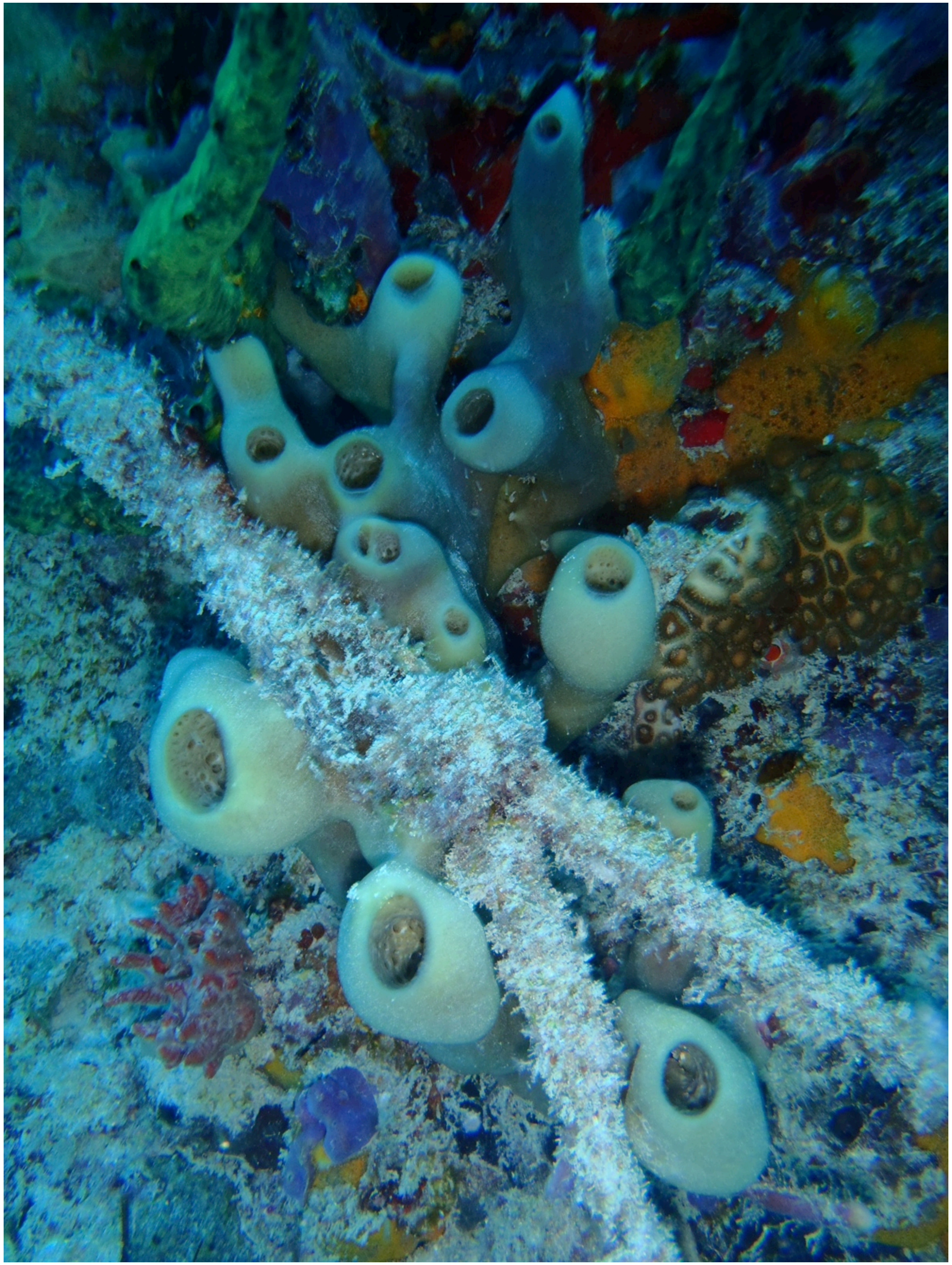

Callyspongia fallax
FAL20
N24° 44.016, W80° 49.590
Coffins Patch Reef
Collected on 03/29/13 by
KML staff

Callyspongia fallax
FAL21
N24° 44.016, W80° 49.590, Coffins Patch Reef
Collected on 03/29/13 by KML staff

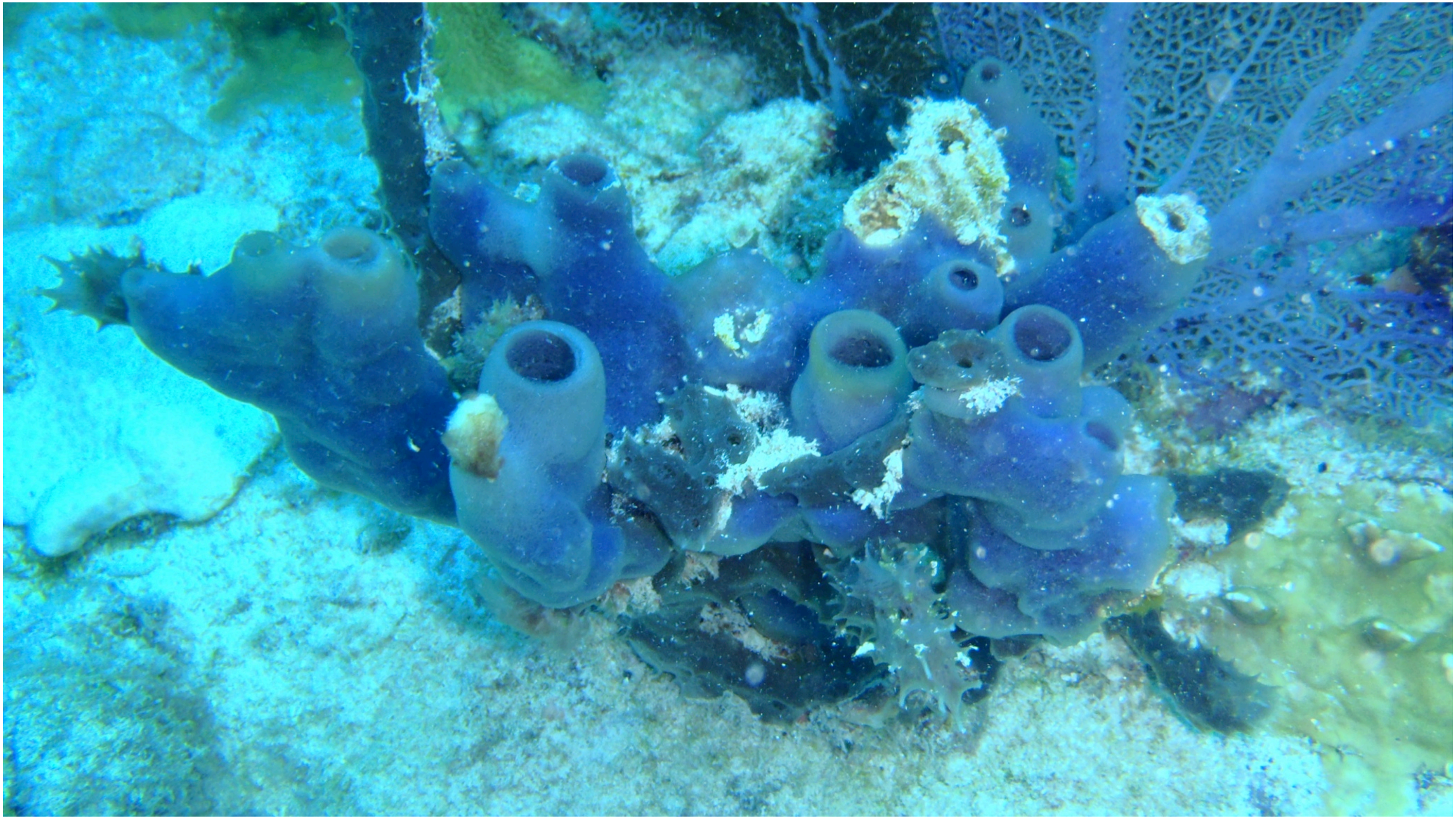

Callyspongia vaginalis
VAG24
N24° 44.016, W80° 49.590, Coffins Patch Reef
Collected on 03/29/13 by KML staff

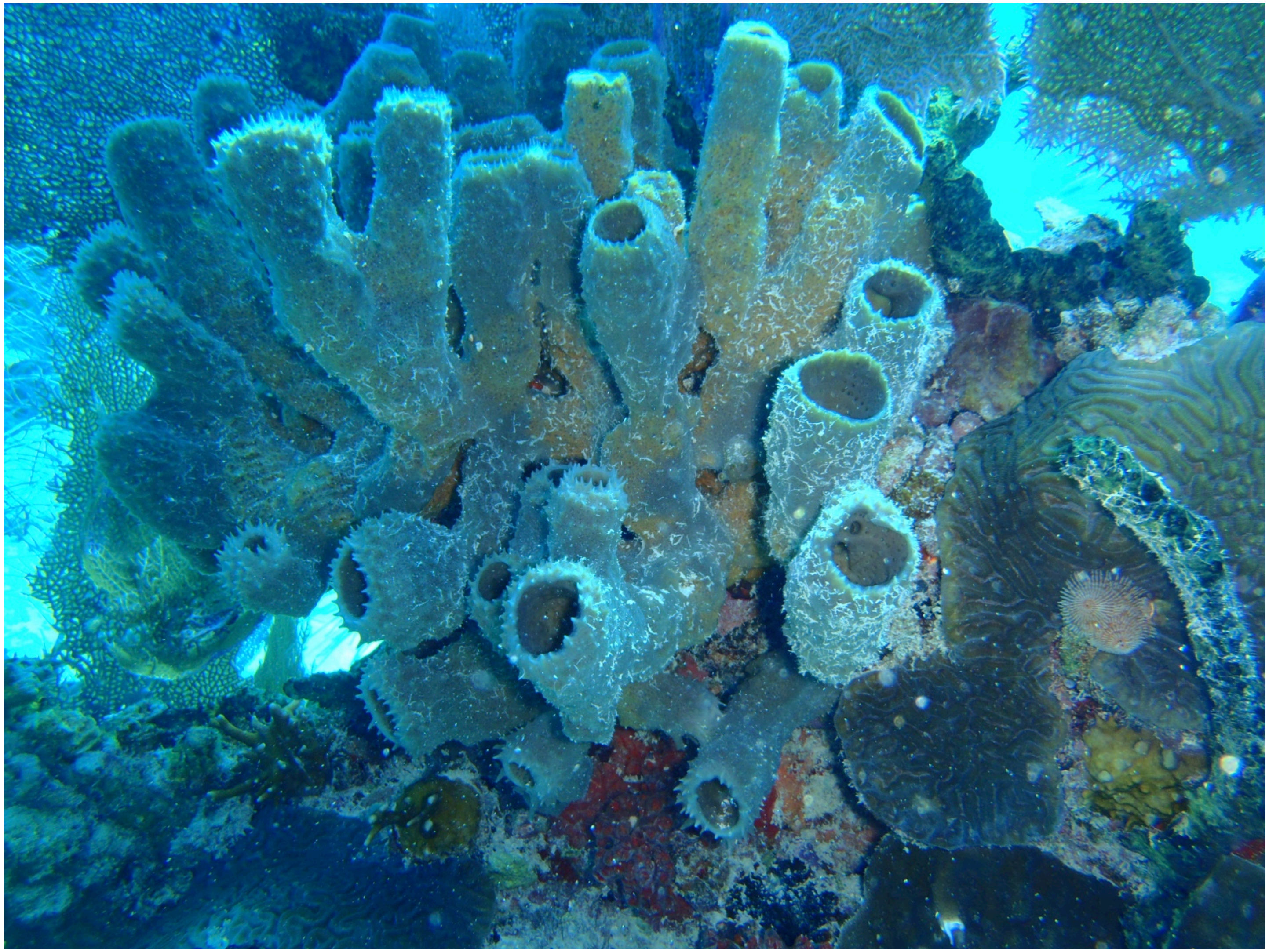

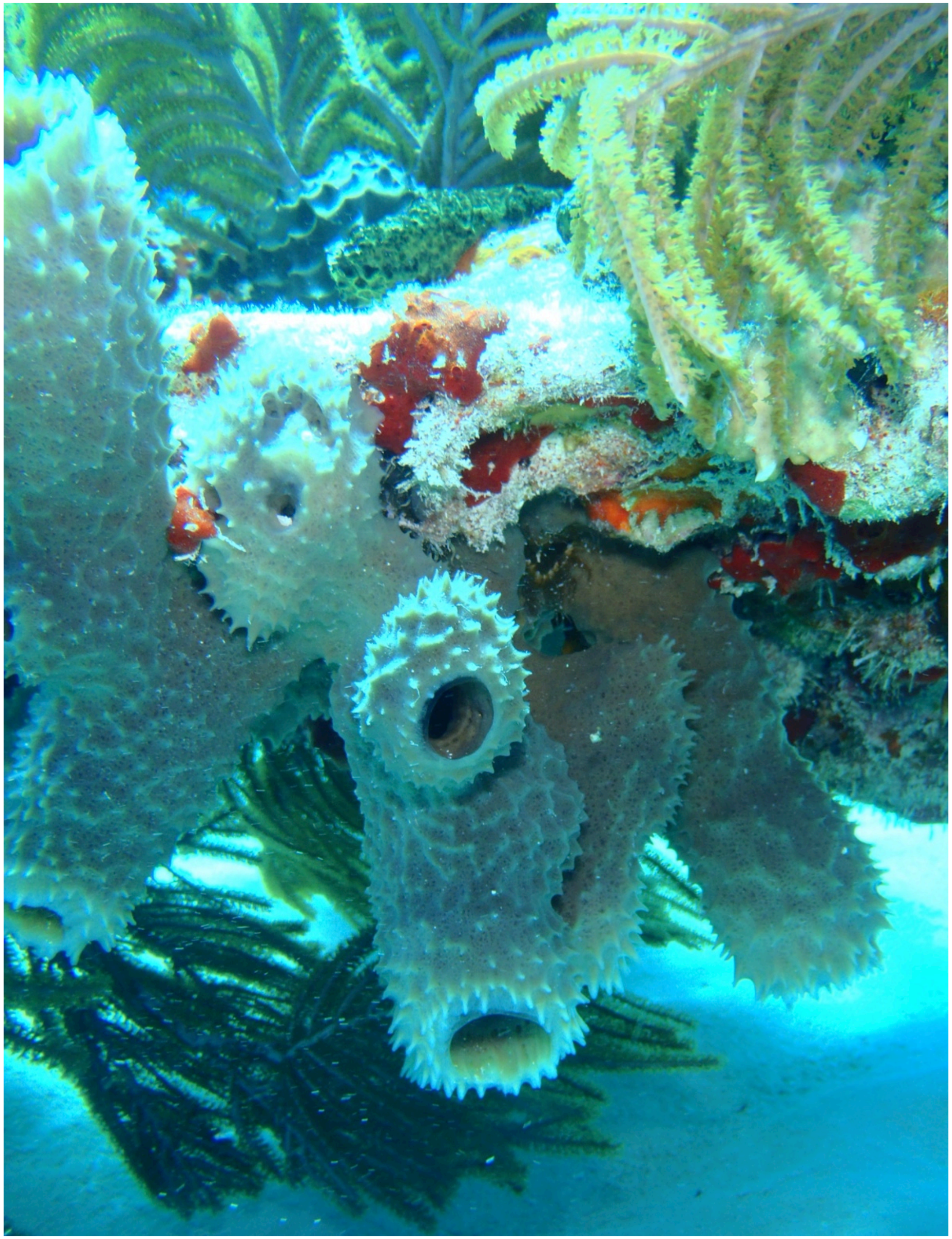

Callyspongia vaginalis
VAG26
N24° 44.016, W80° 49.590
Coffins Patch Reef
Collected on 03/29/13 by
KML staff

Callyspongia vaginalis
VAG39
N24° 44.016, W80° 49.590, Coffins Patch Reef
Collected on 03/29/13 by KML staff

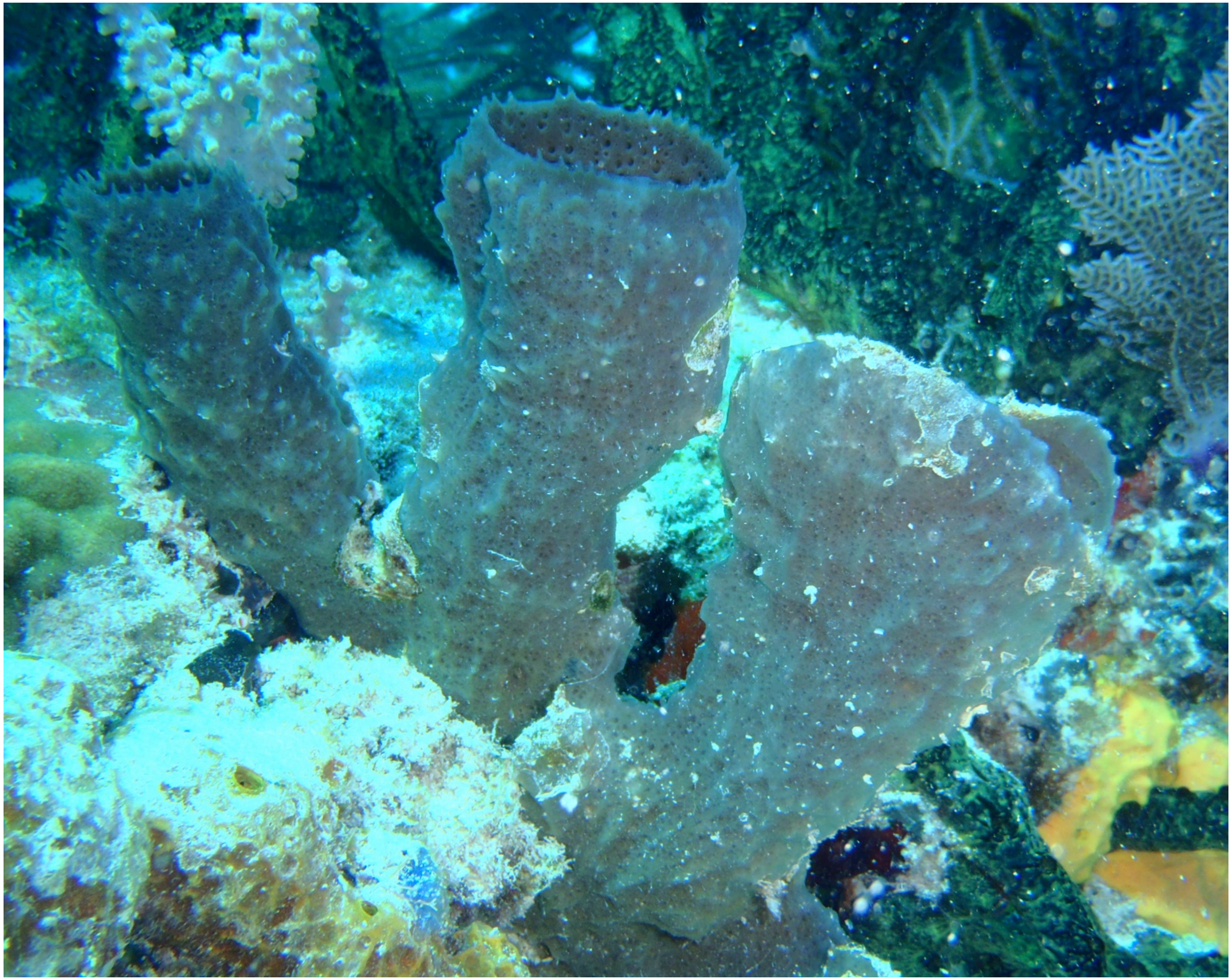

Callyspongia vaginalis
VAG40
N24° 44.016, W80° 49.590, Coffins Patch Reef
Collected on 03/29/13 by KML staff

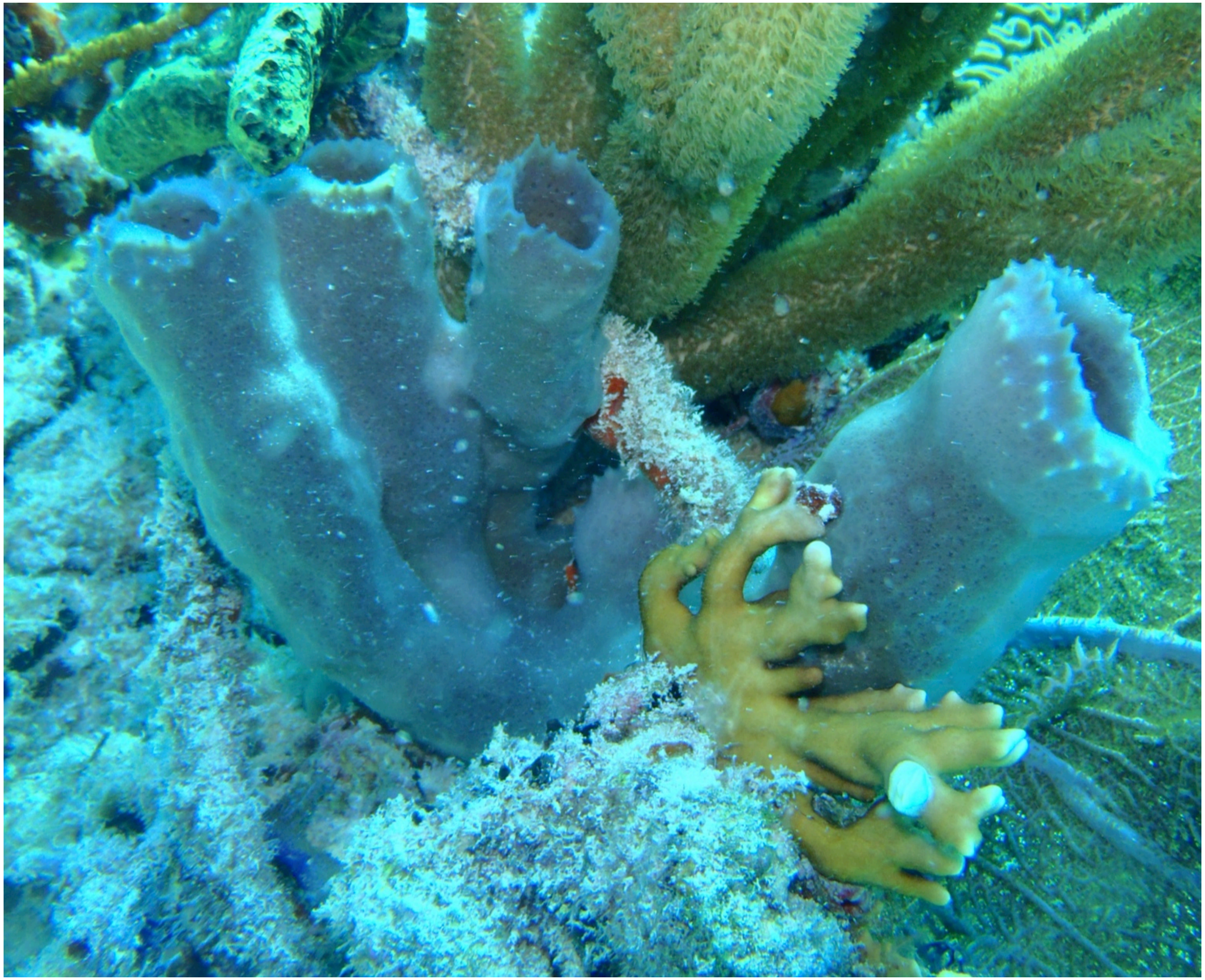

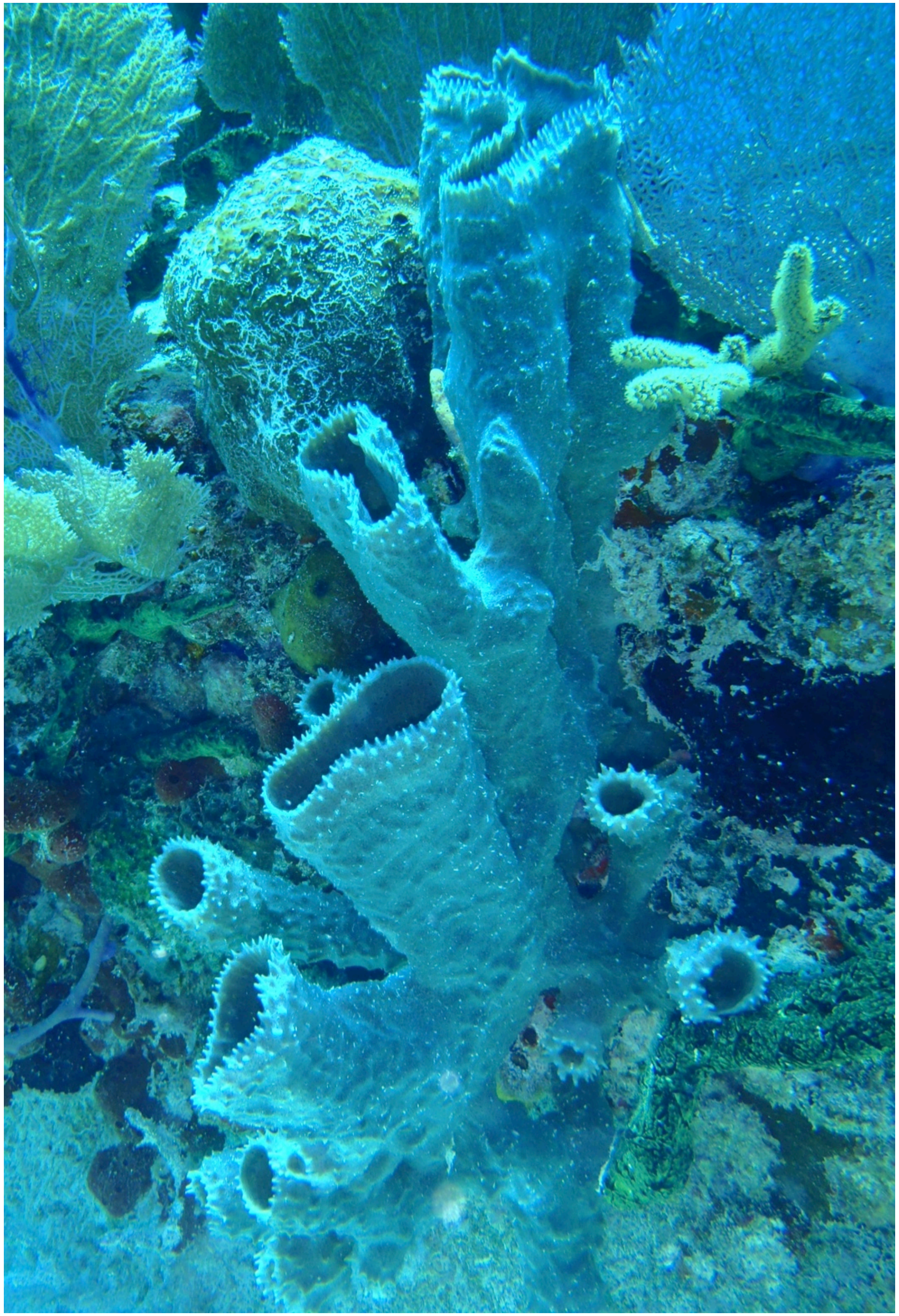

Callyspongia vaginalis
VAG41
N24° 44.016, W80° 49.590
Coffins Patch Reef
Collected on 03/29/13 by
KML staff

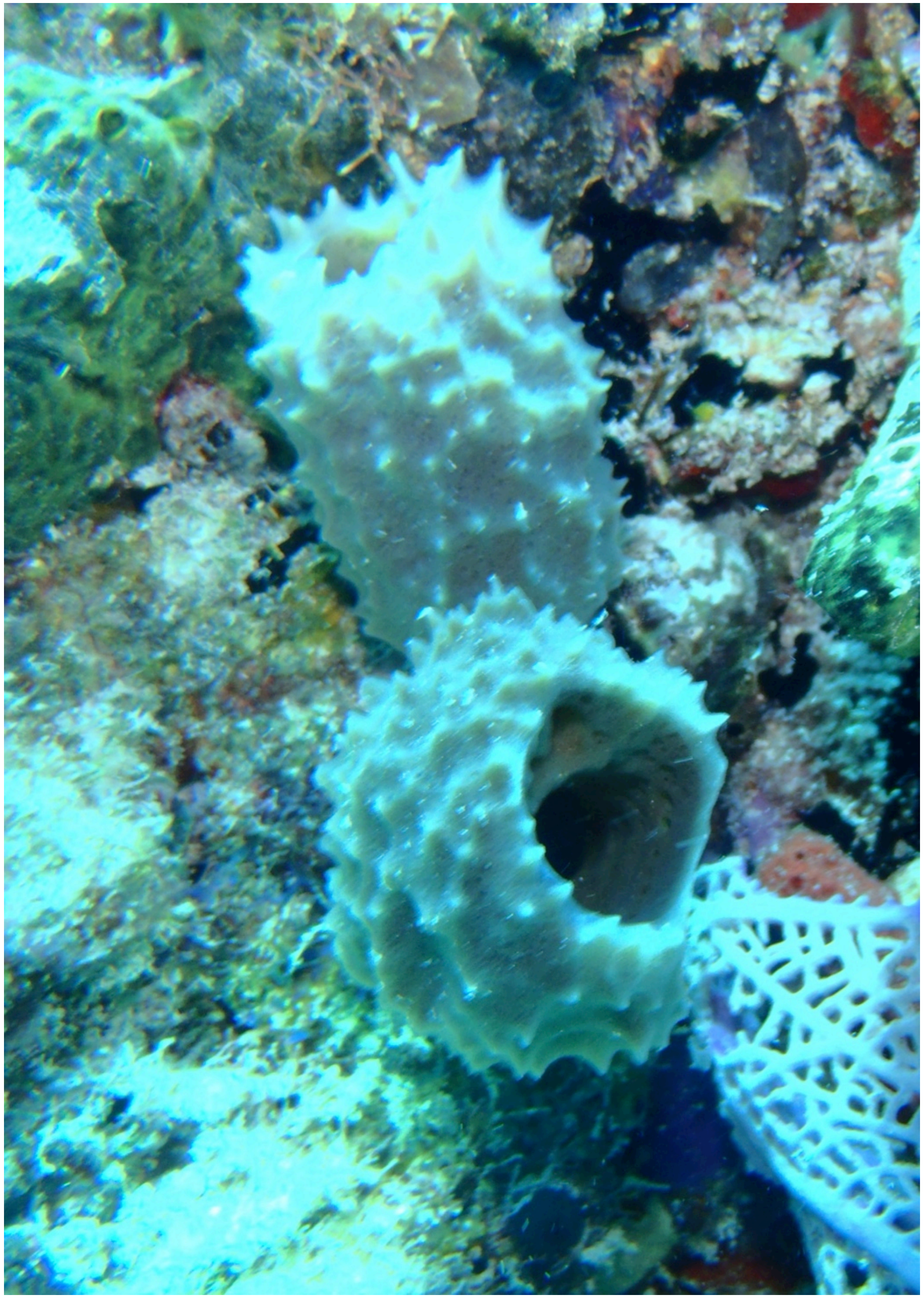

Callyspongia vaginalis
VAG42
N24° 44.016, W80° 49.590
Coffins Patch Reef
Collected on 03/29/13 by
KML staff

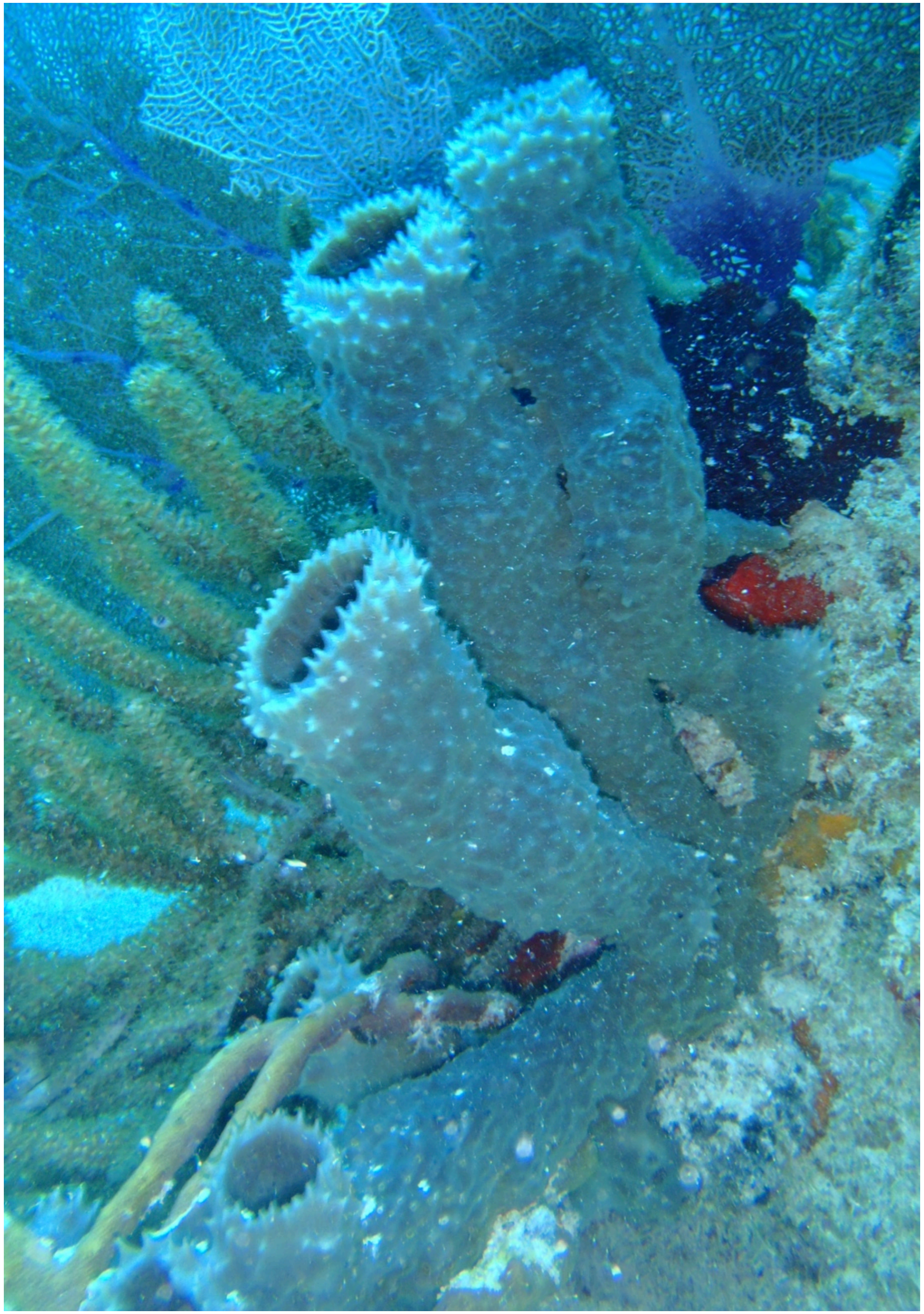

Callyspongia vaginalis
VAG47
N24° 44.016, W80° 49.590
Coffins Patch Reef
Collected on 03/29/13 by
KML staff

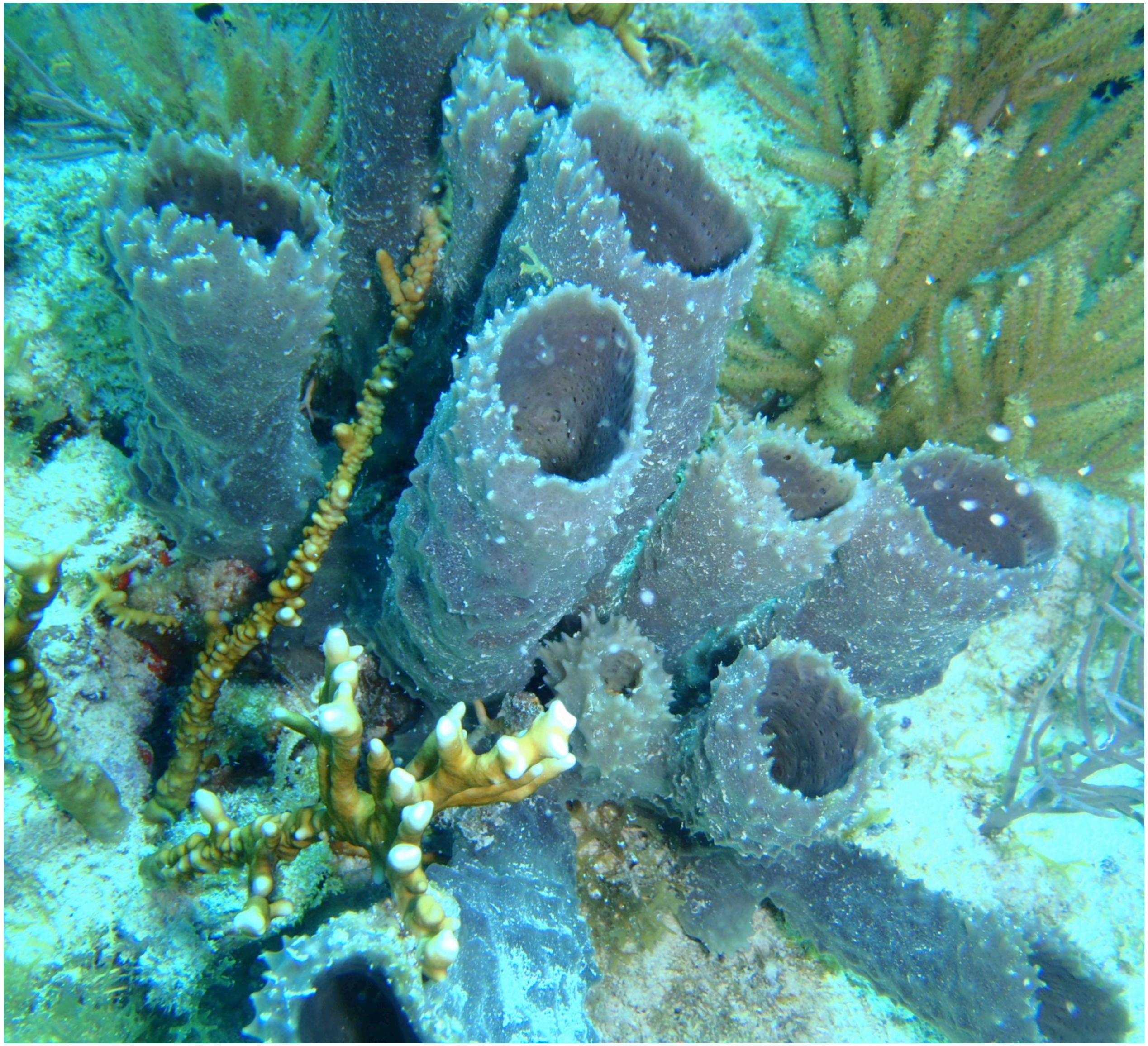

Callyspongia vaginalis
VAG83
N24° 45.953, W80° 45.239
Tennessee Reef
Collected on 04/02/13 by
KML staff

Callyspongia eschrichtii
ESC09
N24° 44.016, W80° 49.590, Coffins Patch Reef
Collected on 03/29/13 by KML staff

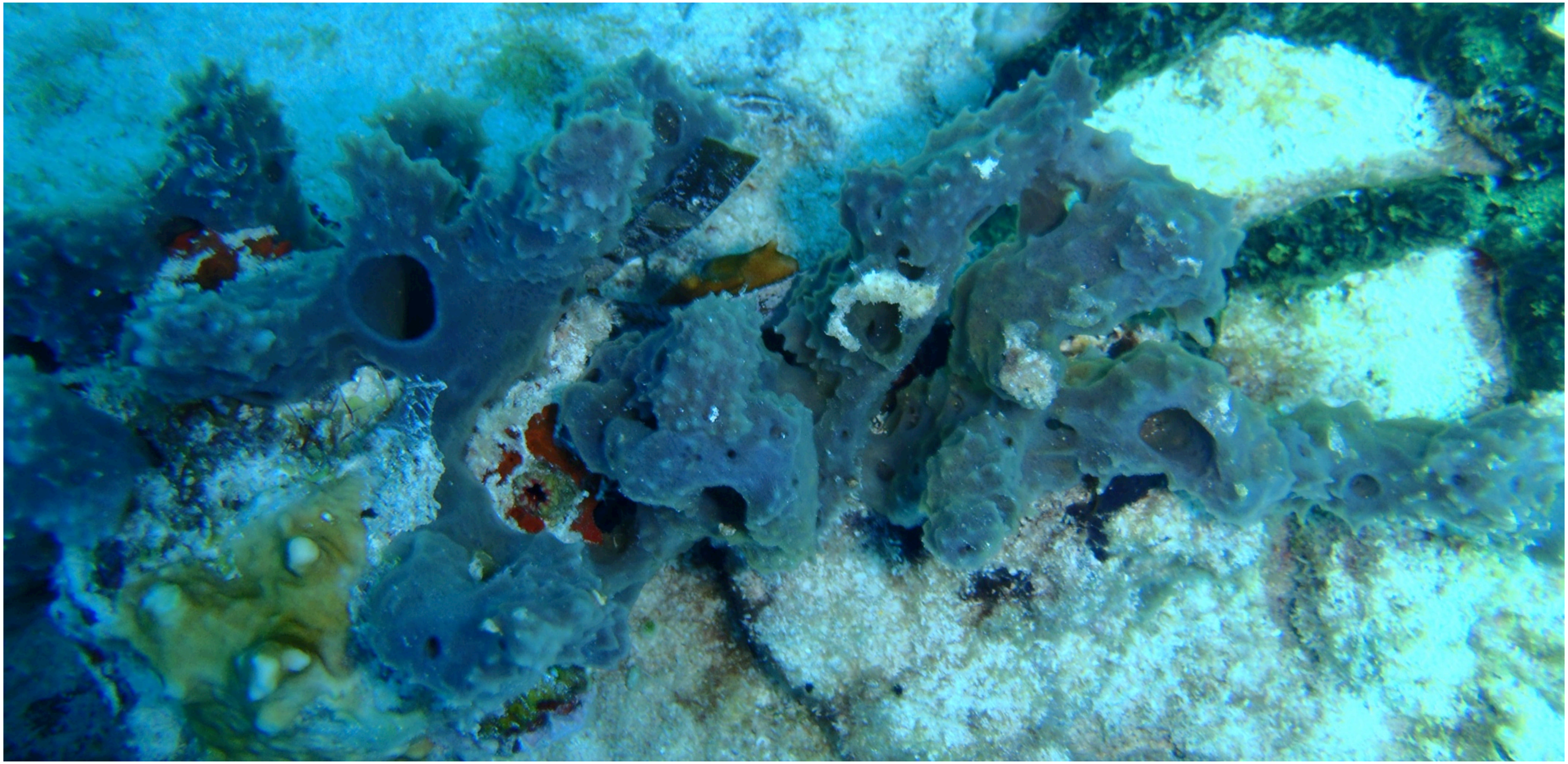

Callyspongia eschrichtii
ESC10
N24° 44.016, W80° 49.590, Coffins Patch Reef
Collected on 03/29/13 by KML staff

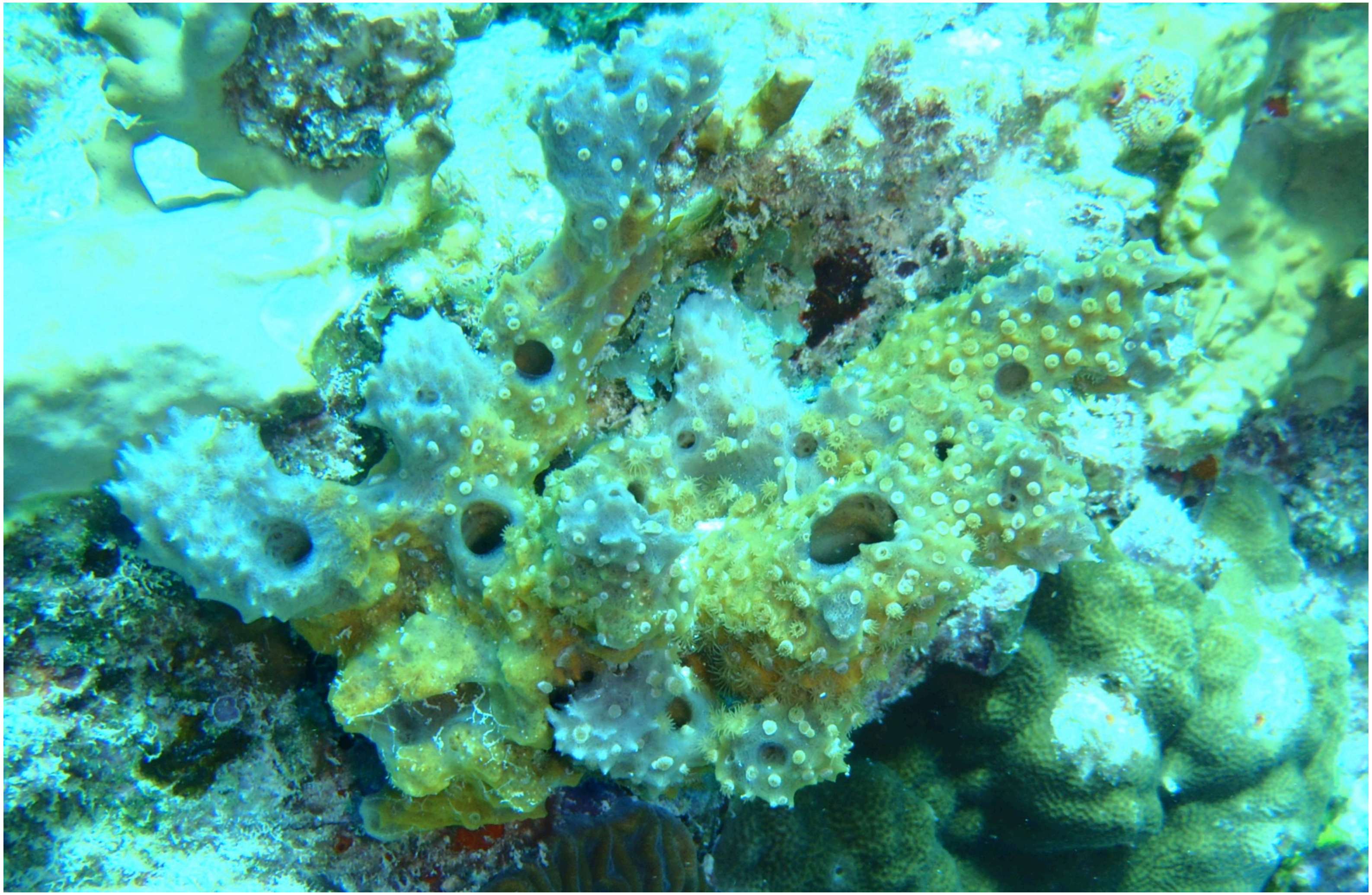

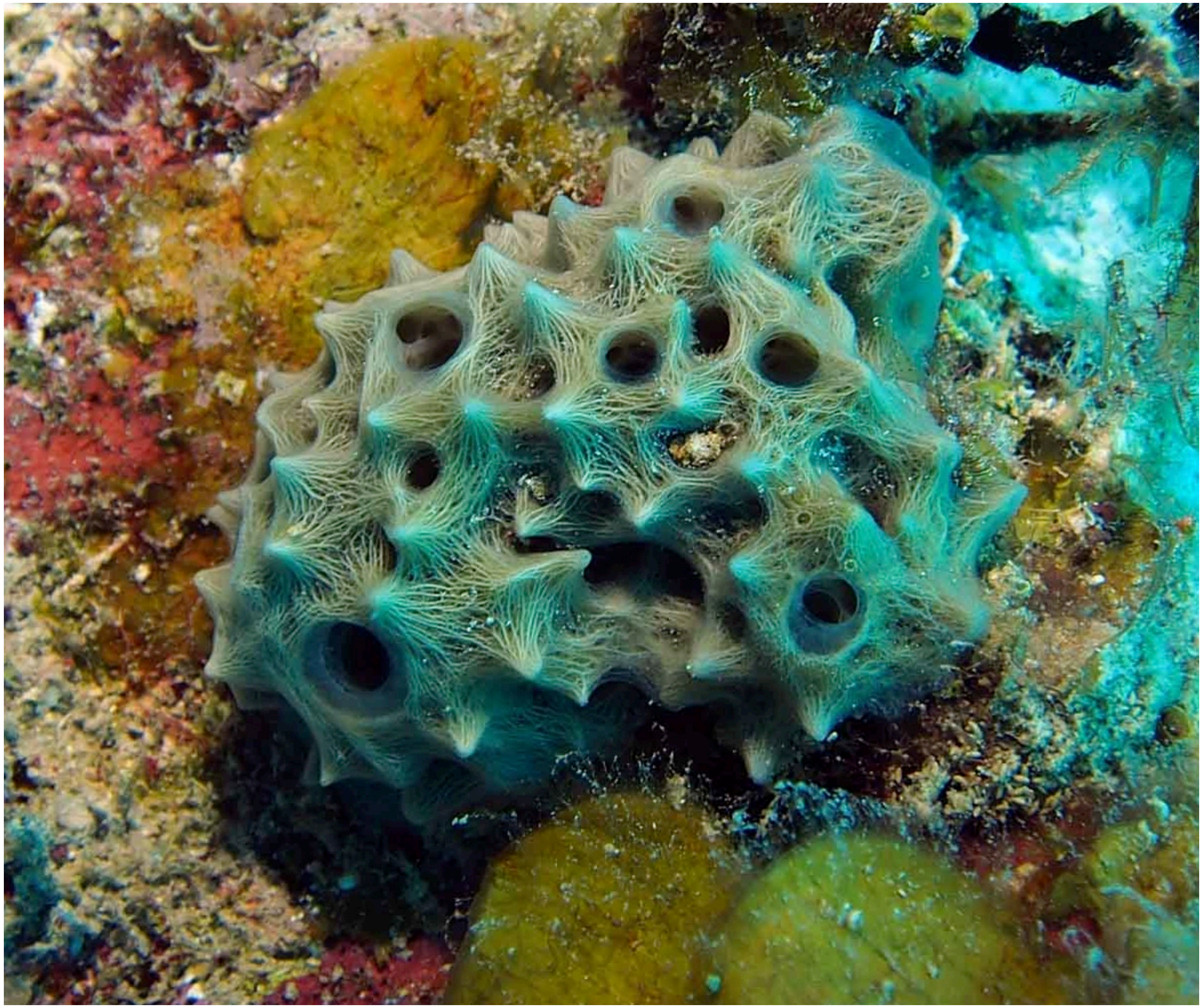

Callyspongia eschrichtii
ESC02 (morphologically synonymous with ESC03, ESC04)
South Acklins Island, Bahamas
Collected on 07/13/13 by J. Pawlik

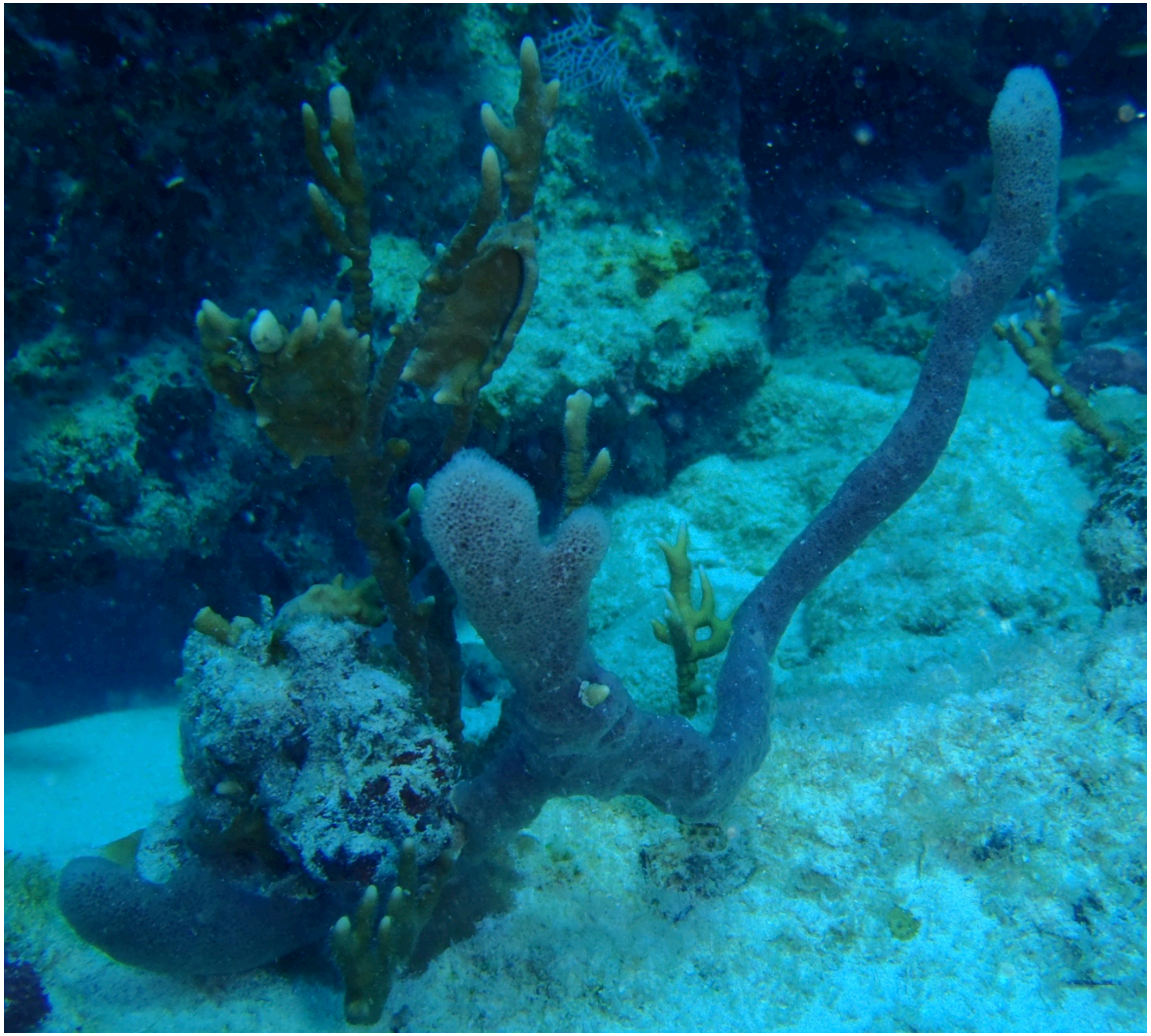

Callyspongia tenerrima
TEN32
N24° 44.016, W80° 49.590
Coffins Patch Reef
Collected on 03/29/13 by
KML staff

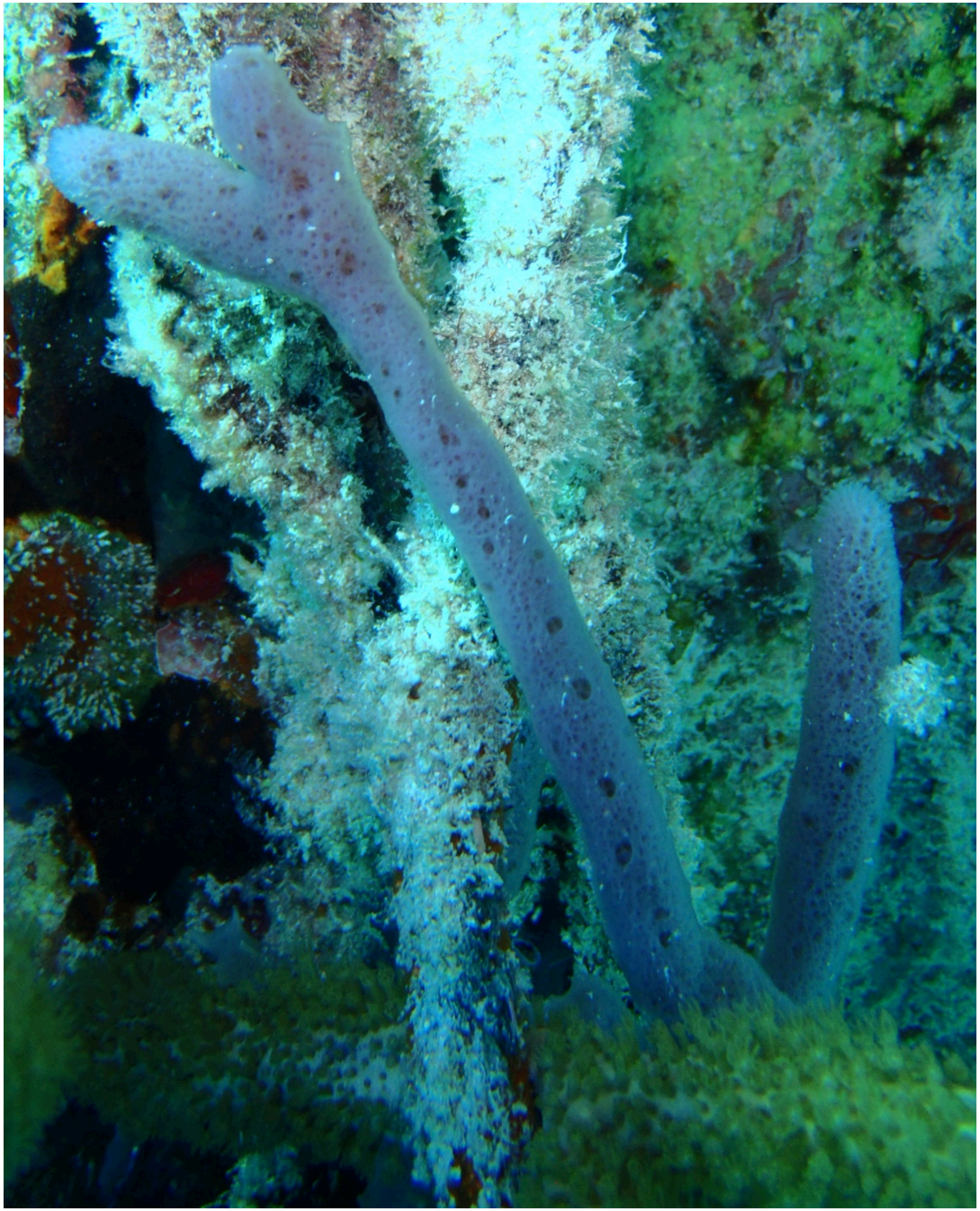

Callyspongia tenerrima
TEN34
N24° 44.016, W80° 49.590
Coffins Patch Reef
Collected on 03/29/13 by
KML staff

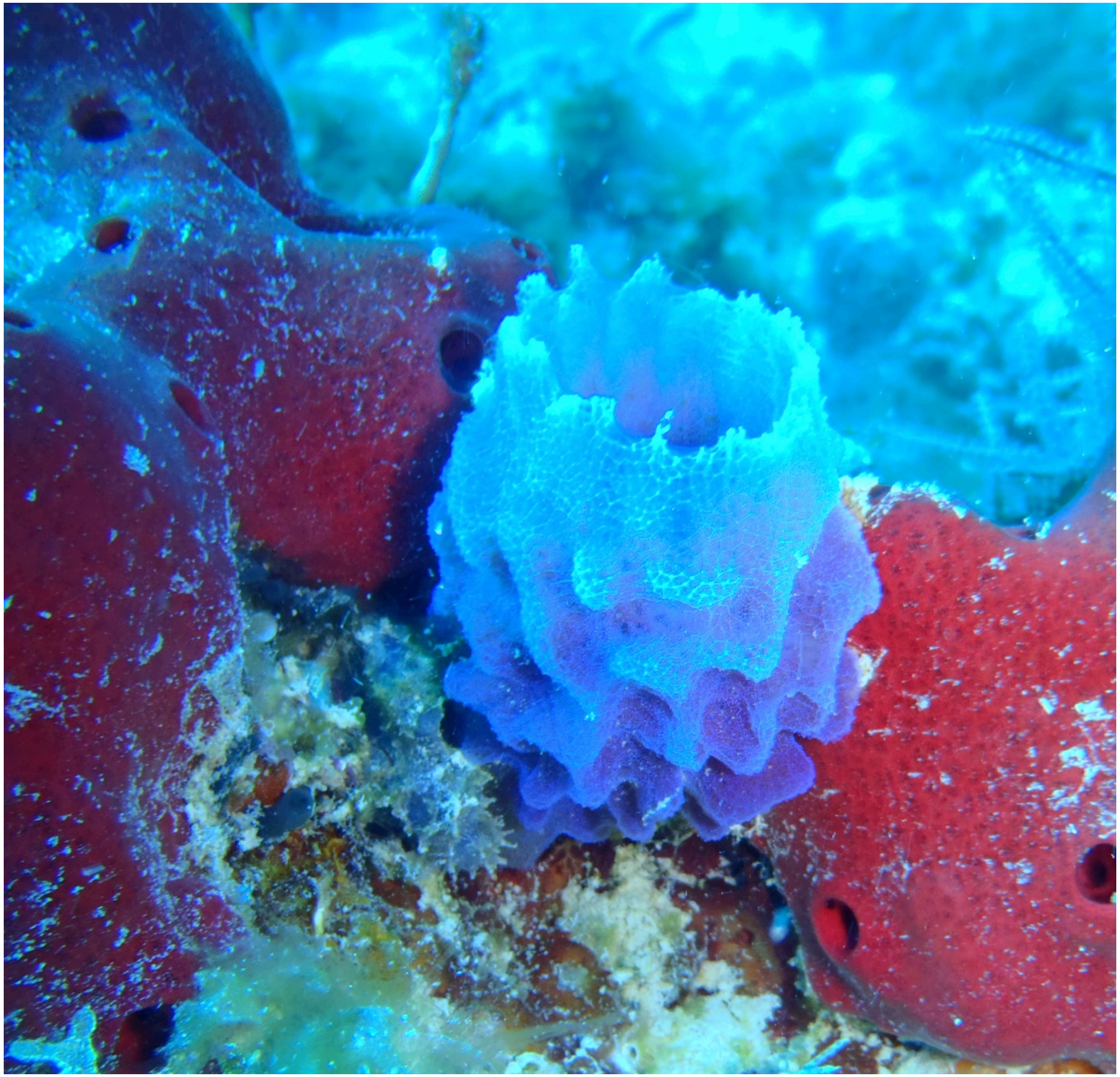

Callyspongia plicifera
PLI64
N24° 45.953, W80° 45.239
Tennessee Reef
Collected on 04/02/13 by
KML staff

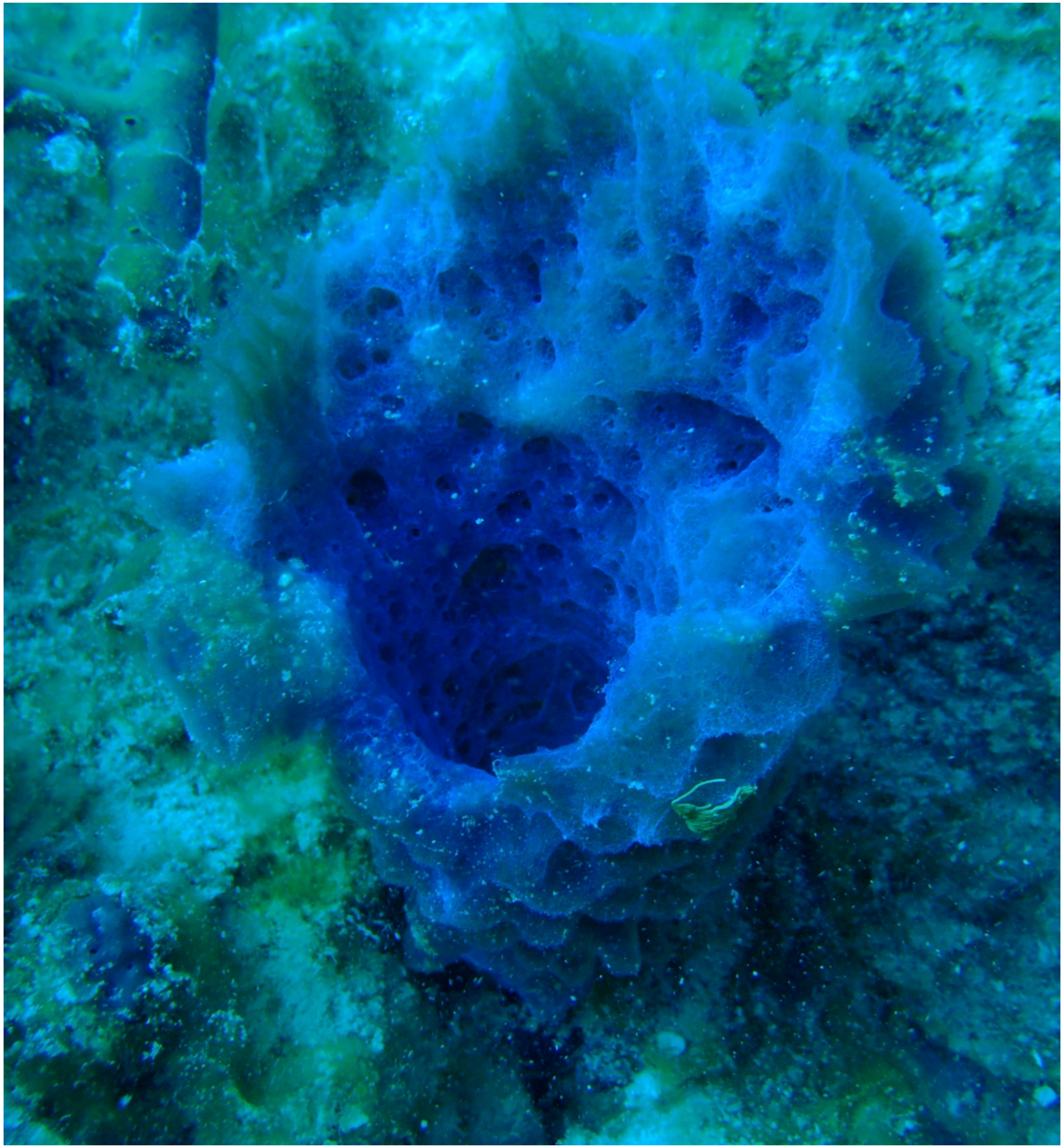

Callyspongia plicifera
PLI65
N24° 45.953, W80° 45.239
Tennessee Reef
Collected on 04/02/13 by
KML staff

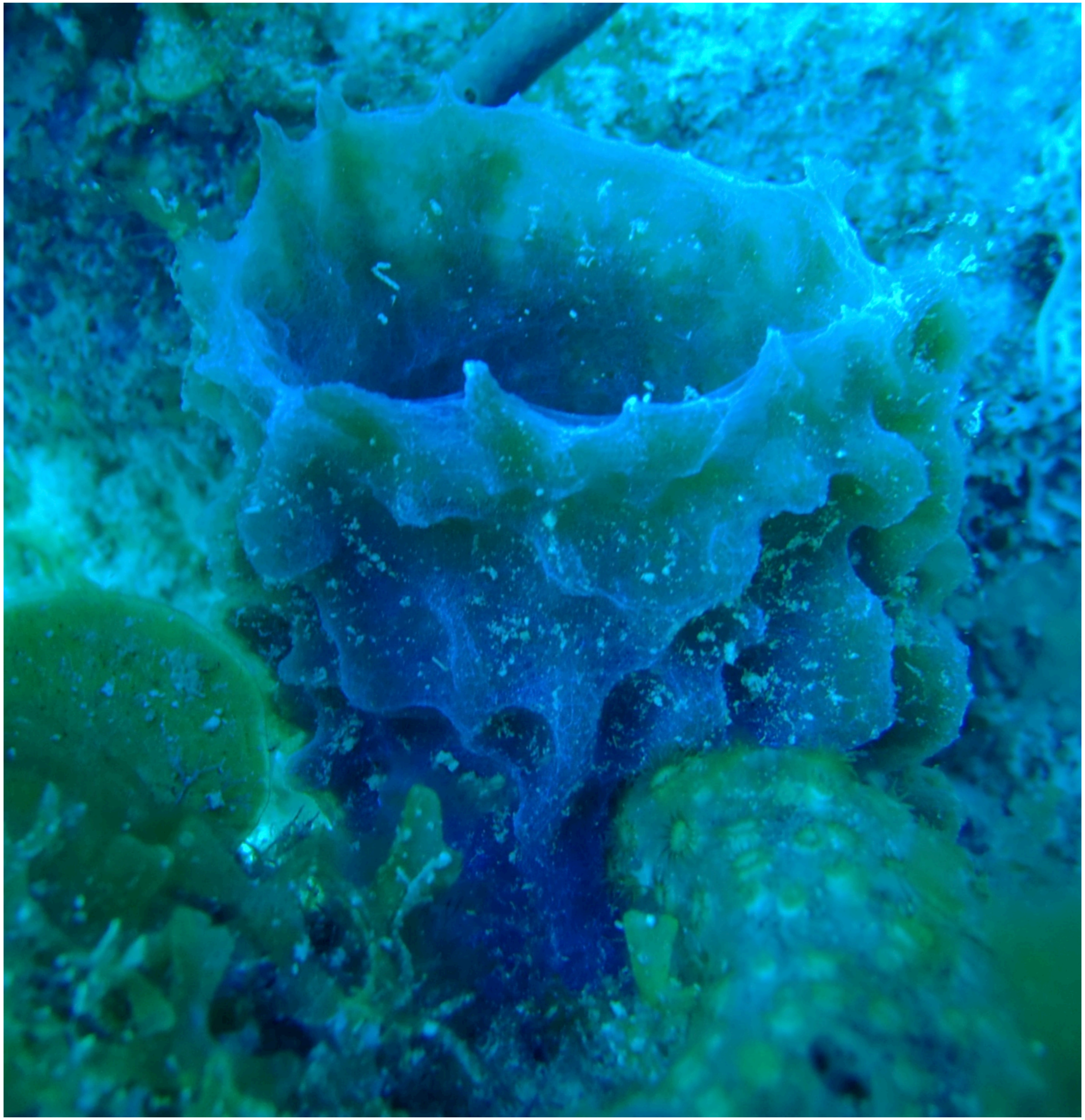

Callyspongia plicifera
PLI66
N24° 45.953, W80° 45.239
Tennessee Reef
Collected on 04/02/13 by
KML staff

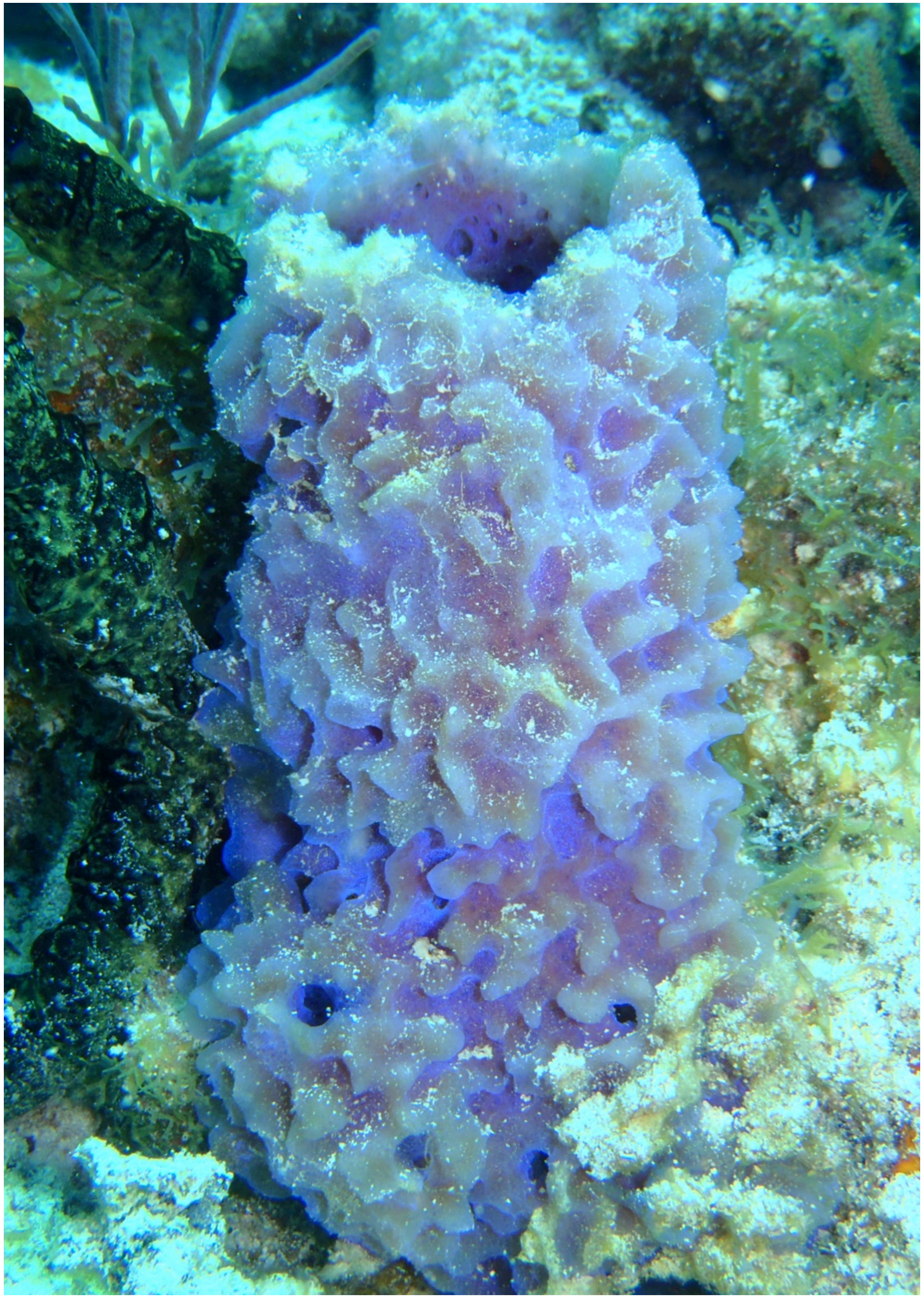

Callyspongia plicifera
PLI67
N24° 45.953, W80° 45.239
Tennessee Reef
Collected on 04/02/13 by
KML staff

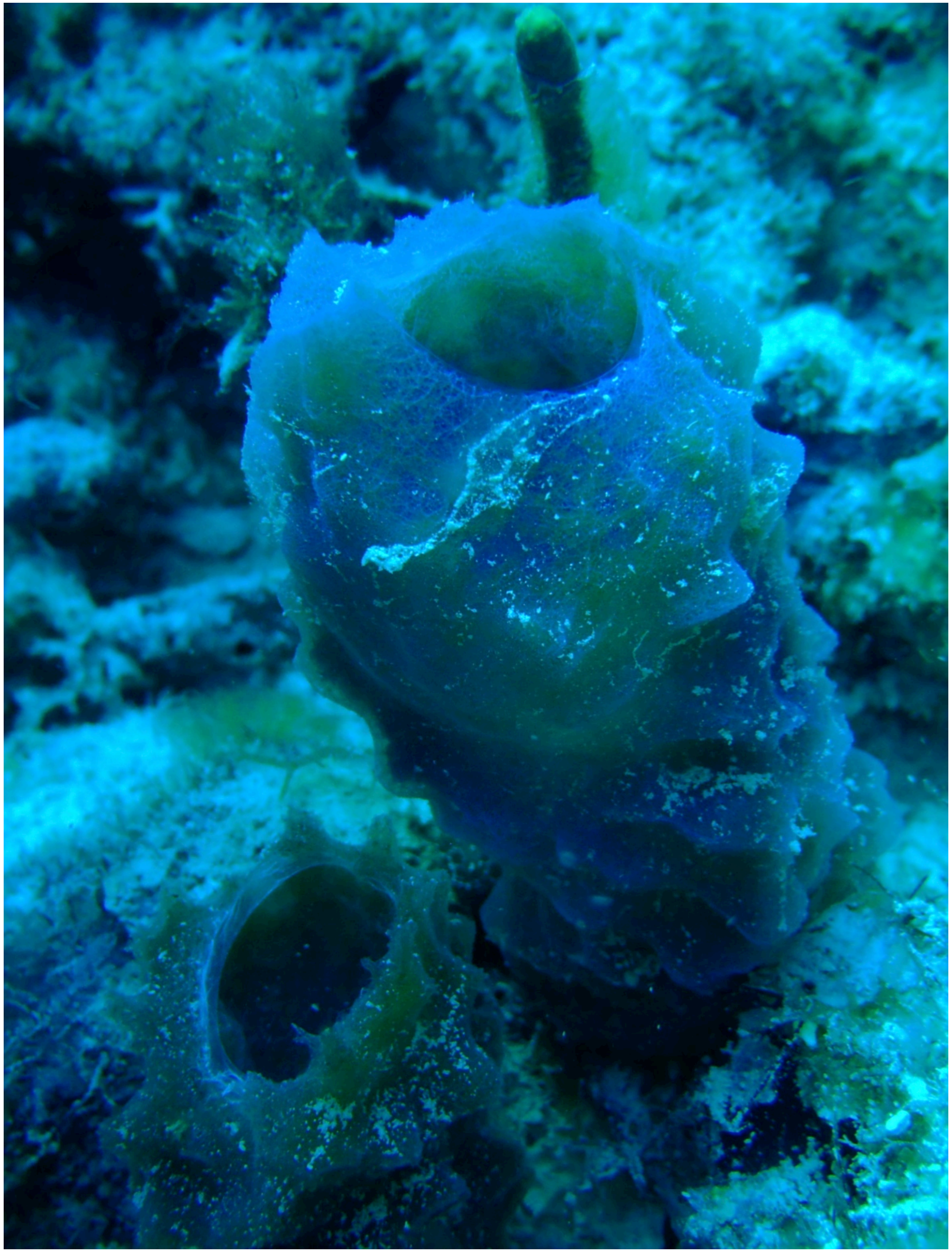

Callyspongia plicifera
PLI68
N24° 45.953, W80° 45.239
Tennessee Reef
Collected on 04/02/13 by
KML staff

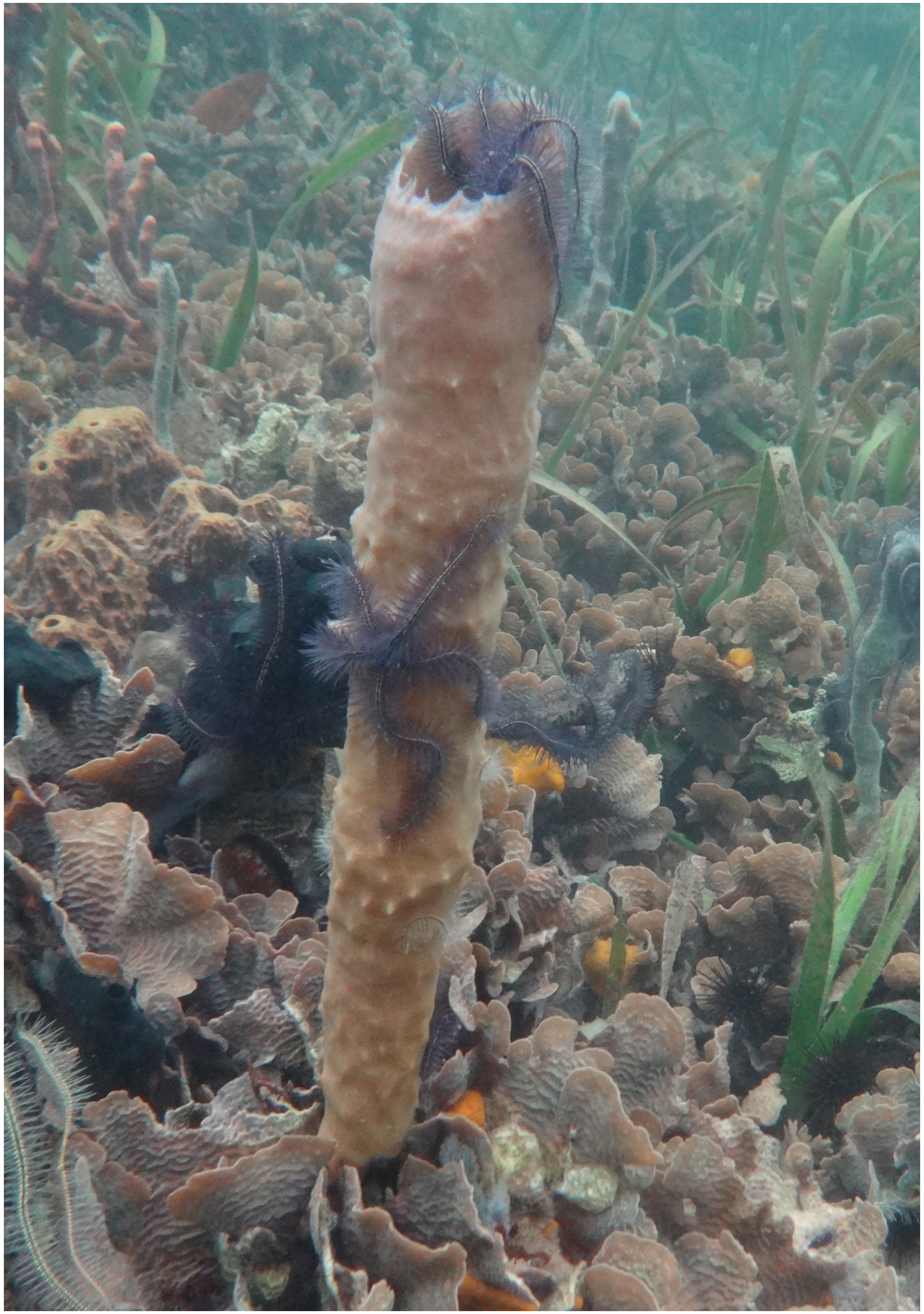

Callyspongia longissima
LON01
N 9° 21 1.48 W 82° 15 27.31
Bocas del Toro, Panama
Collected on 06/24/13 by M. DeBiasse
