# Supplementary material for: Discordance between morphological and molecular species boundaries among Caribbean species of the reef sponge Callyspongia
Source: Ecol Evol. 2015 Jan 13;5(3):663–75. doi: 10.1002/ece3.1381 (PMC4328770; doi:10.1002/ece3.1381)
Supplement: Supplementary file 4 [file ece30005-0663-sd4.pdf]

Supplementary Tables and Figures

Table 1 Primer sequences and expected amplicon size for gene regions used in this study.

Gene name	Primer sequence (5' – 3')	Amplicon size
5' *COI*[1]	F: ATATAATGTTATAGTGACAGCTCATGC<br>R: ATTGGAACTATCGGCTCCGGG	350 bp
3' *COI*[1]	F: TGGTTATTTGGGGATGGTATATGC<br>R: TAACAATACCCMGAWATTTTCCC	350 bp
28S[2]	5F: TAGGTCGACCCGCTGCCYTTAAGC<br>300R: CAACTTTCCCTCACGGTACTT	400 bp
Filamin[3] (*fil*)	F: CAGTACAATATAAGCATGGTCCCTCG<br>R: GTATAGGCTCACACAGTCCTTCCC	230 bp
Macrophage expressed protein[3] (*mep*)	F: CTTTCTCTATAAGCACTAATATTGG<br>R: AGTAGTCATAGCAAATGAGAGAGG	210 bp

[1]Designed for this study, [2]Redmond *et al.* (2011), [3]DeBiasse *et al.* (2014)

Table 2 *COI* sequences downloaded from GenBank used in Figure 3

Species	Accession No.	Reference
Callyspongia armigera	EF519578	Erpenbeck *et al.* (2007)
Callyspongia fallax	JN242192	Redmond *et al.* (2011)
Callyspongia fallax	JN242193	Redmond *et al.* (2011)
Callyspongia fallax	GQ415417	López-Legentil *et al.* (2010)
Callyspongia fallax	GQ415416	López-Legentil *et al.* (2010)
Callyspongia plicifera	NC010206	Lavrov *et al.* (2008)
Callyspongia plicifera	EU23477	Kayal and Lavrov (2008)
Callyspongia vaginalis	GQ415415	López-Legentil *et al.* (2010)
Callyspongia vaginalis	GQ415414	López-Legentil *et al.* (2010)
Callyspongia vaginalis	GQ415413	López-Legentil *et al.* (2010)
Callyspongia vaginalis	GQ415412	López-Legentil *et al.* (2010)
Callyspongia vaginalis	EF095182	Itskovich *et al.* (2007)
Callyspongia vaginalis	EF519577	Erpenbeck *et al.* (2007)
Callyspongia vaginalis	EF519579	Erpenbeck *et al.* (2007)
Callyspongia vaginalis	EF519581	Erpenbeck *et al.* (2007)
Callyspongia vaginalis	EF519580	Erpenbeck *et al.* (2007)
Haliclona cinerea	JN242198	Redmond *et al.* (2011)
Haliclona coerulea	EF519619	Erpenbeck *et al.* (2007)
Haliclona implexiformia	EF519624	Erpenbeck *et al.* (2007)
Haliclona simulans	JN242201	Redmond *et al.* (2011)
Haliclona toxius	JN242206	Redmond *et al.* (2011)
Haliclona tubifera	EF519624	Erpenbeck *et al.* (2007)

Tables 3A-E Mean Kimura 2 Parameter genetic distances within and among species for each gene region. Italicized values are those estimated within species. Dashes indicate species for which only one individual was sequenced.

**A: 5'COI**	ARM	ESC	FAL	LON	PLI	VAG
ARM	*0.018*					
ESC	0.017	*0.000*				
FAL	0.268	0.273	*0.004*			
LON	0.011	0.012	0.273	-		
PLI	0.260	0.264	0.098	0.264	-	
VAG	0.020	0.017	0.265	0.020	0.260	*0.015*

**B: 3'COI**	ARM	ESC	FAL	LON	PLI	TEN	VAG
ARM	*0.016*						
ESC	0.013	*0.006*					
FAL	0.273	0.275	*0.002*				
LON	0.010	0.005	0.275	-			
PLI	0.271	0.270	0.117	0.270	*0.000*		
TEN	0.261	0.261	0.096	0.264	0.101	-	
VAG	0.018	0.018	0.274	0.019	0.276	0.259	*0.015*

**C: 28S**	ARM	ESC	FAL	LON	PLI	TEN	VAG
ARM	*0.000*						
ESC	0.000	*0.000*					
FAL	0.209	0.209	*0.001*				
LON	0.000	0.000	0.209	-			
PLI	0.171	0.171	0.092	0.171	*0.000*		
TEN	0.171	0.171	0.090	0.171	0.066	*0.000*	
VAG	0.000	0.000	0.171	0.000	0.171	0.171	*0.000*

**D: FIL**	ARM	ESC	FAL	LON	VAG
ARM	*0.026*				
ESC	0.026	*0.026*			
FAL	0.027	0.019	*0.024*		
LON	0.021	0.023	0.020	*0.024*	
VAG	0.024	0.025	0.023	0.022	*0.022*

**E: MEP**	ARM	ESC	FAL	LON	VAG
ARM	*0.029*				
ESC	0.026	*0.000*			
FAL	0.026	0.000	*0.000*		
LON	0.024	0.024	0.024	*0.000*	
VAG	0.028	0.026	0.026	0.026	*0.027*

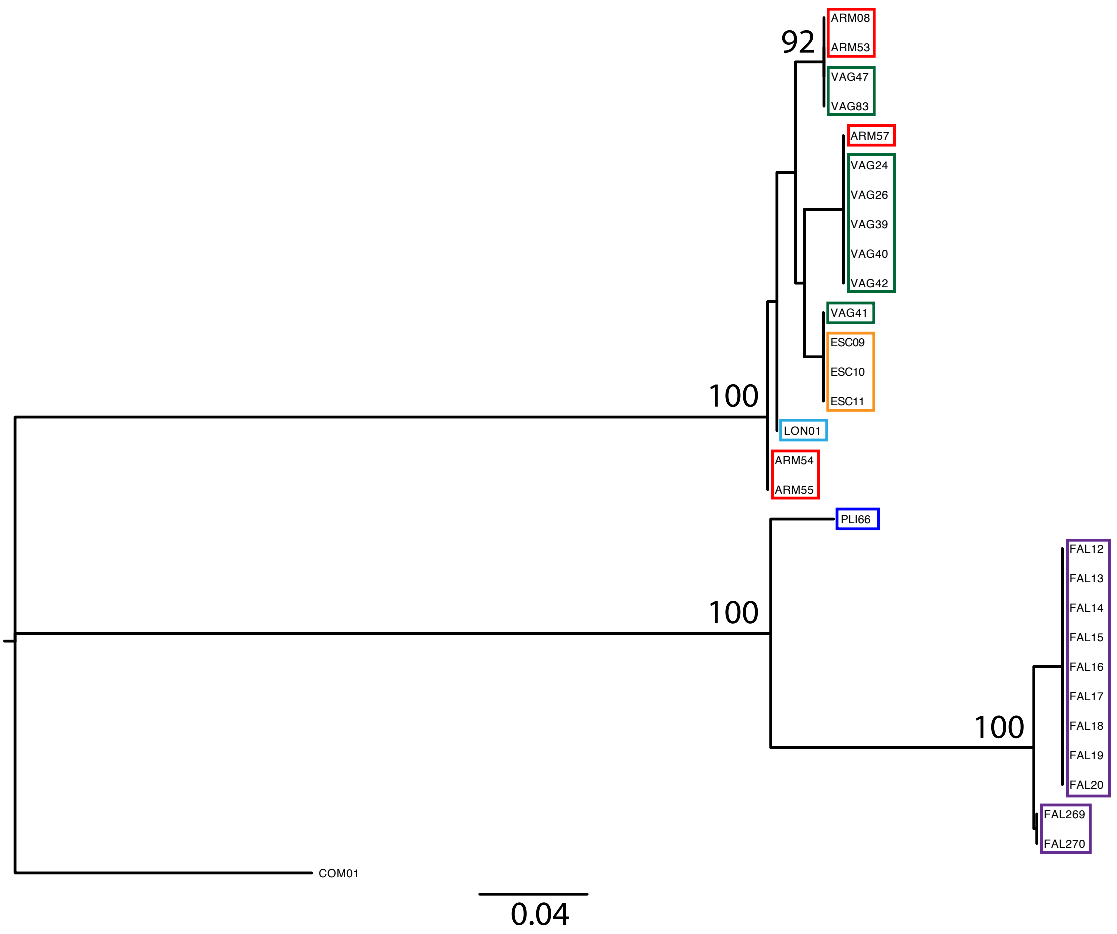

Figure 1 Maximum likelihood tree estimated in PAUP* using the 5' region of the *COI* gene. Colored boxes indicate each species corresponding to the legend in Figure 1. Support values from 100 bootstrap replicates appear at the nodes when greater than 50. The tree is rooted with *Amphimedon compressa* (accession number NC_010201).

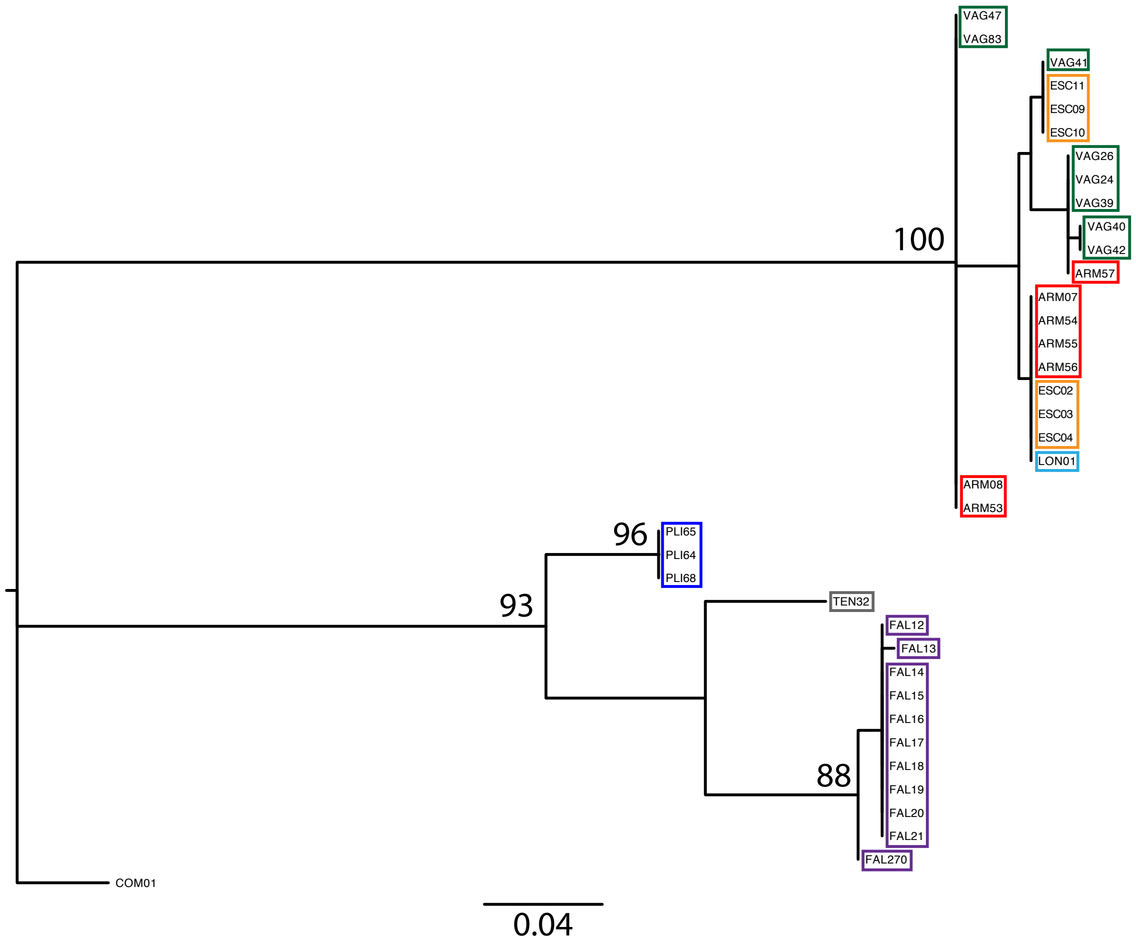

Figure 2 Maximum likelihood tree estimated in PAUP* using the 3' region of the *COI* gene. Colored boxes indicate each species corresponding to the legend in Figure 1. Support values from 100 bootstrap replicates appear at the nodes when greater than 50. The tree is rooted with *Amphimedon compressa* (accession number NC_010201).

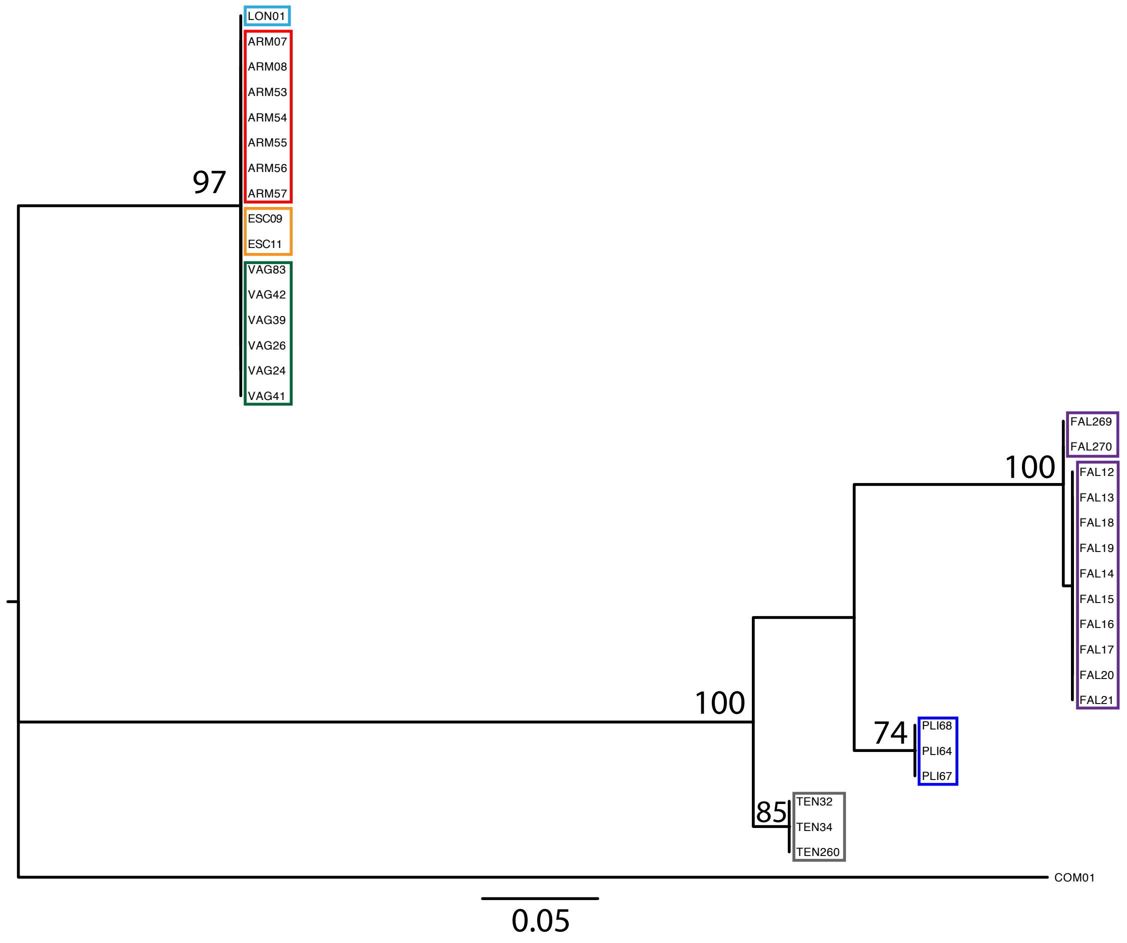

Figure 3 Maximum likelihood tree estimated in PAUP* using the D1 region of the 28S gene. Colored boxes indicate each species corresponding to the legend in Figure 1. Support values from 100 bootstrap replicates appear at the nodes when greater than 50. The tree is rooted with *Amphimedon compressa* (accession number JN178945).

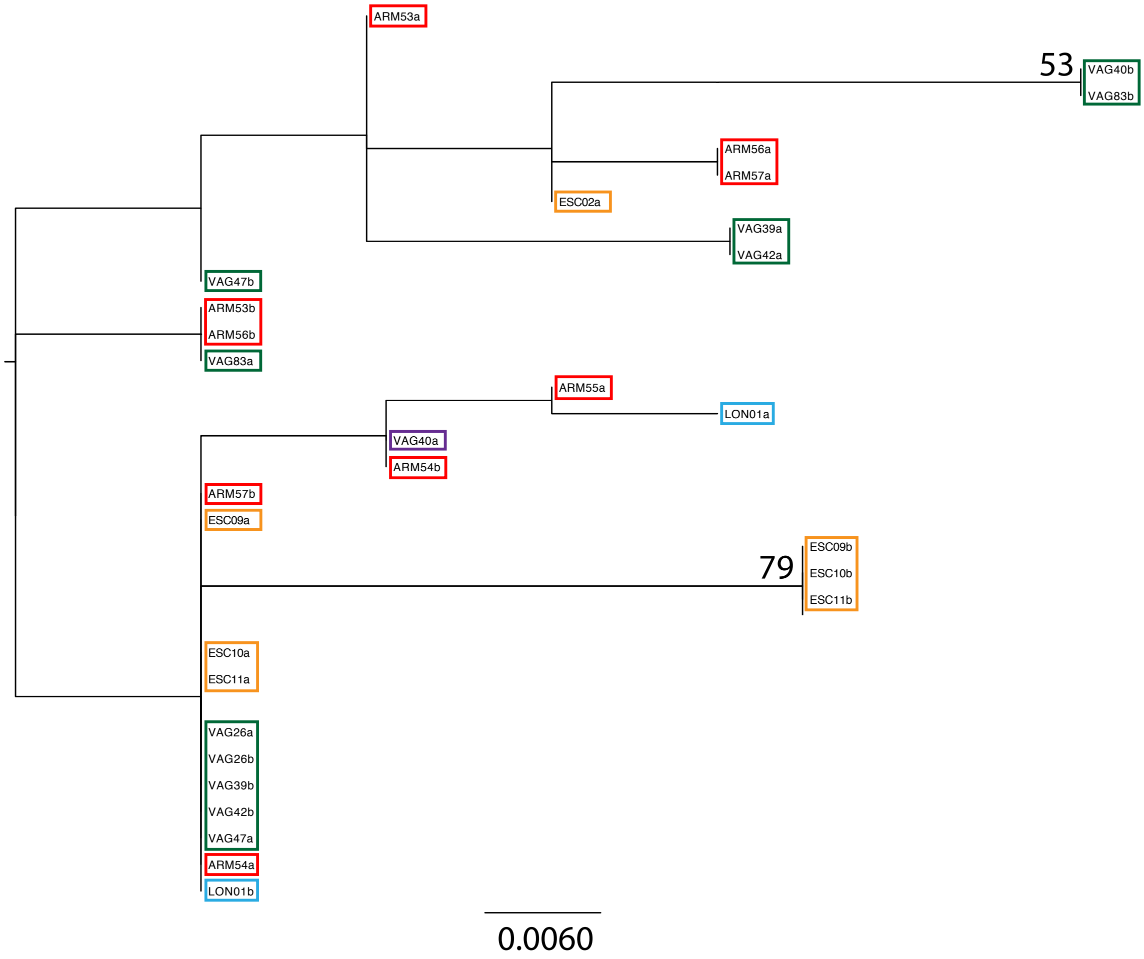

Figure 4 Maximum likelihood tree estimated in PAUP* using a portion of the filamin gene. Colored boxes indicate each species corresponding to the legend in Figure 1. No bootstrap support values were greater than 50. The tree is rooted at its midpoint.

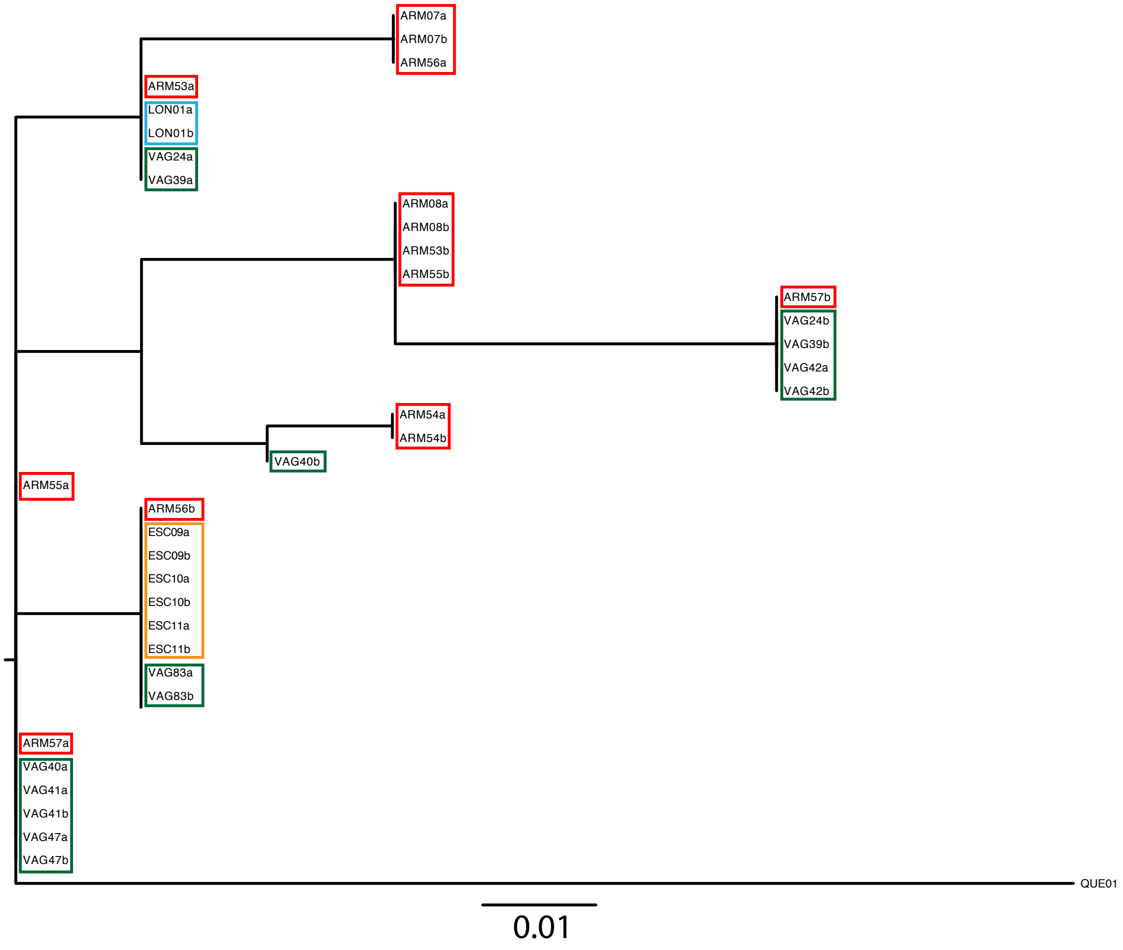

Figure 5 Maximum likelihood tree estimated in PAUP* using a portion of the macrophage expressed protein gene. Colored boxes indicate each species corresponding to the legend in Figure 1. No bootstrap support values were greater than 50. The tree is rooted with *Amphimedon queenslandica* (accession number GCF_000090795).
